# Benchmarking large language models against practicing clinicians on psychopathological assessment

**DOI:** 10.1038/s41746-026-02852-7

**Published:** 2026-07-07

**Authors:** Esra Lenz, Joonas Naamanka, Wolfgang Trabert, Ronald Bottlender, Berend Malchow, Andreas Meyer-Lindenberg, Tobias Gradinger, Emanuel Schwarz

**Affiliations:** 1 Hector Institute for Artificial Intelligence in Psychiatry, Central Institute of Mental Health, Medical Faculty Mannheim, Heidelberg University, Mannheim, Germany; 2 Department of Psychiatry and Psychotherapy, Central Institute of Mental Health, Medical Faculty Mannheim, Heidelberg University, Mannheim, Germany; 3 SleepWell Research Program, Faculty of Medicine, University of Helsinki, Helsinki, Finland; 4 Association for Methodology and Documentation in Psychiatry (AMDP), Homburg/Saar, Germany; 5 LWL-Kliniken Lippstadt und Warstein, Warstein, Germany; 6 Dept. of Psychiatry and Psychotherapy, University Medical Center Goettingen, Goettingen, Germany; 7 German Center for Mental Health (DZPG), Partner Site Mannheim – Heidelberg, Ulm, Germany

**Keywords:** Diseases, Health care, Medical research, Psychology, Psychology

## Abstract

Psychiatry’s reliance on language makes LLMs a natural tool for psychopathological assessment, yet structured, item-level assessments from psychiatric clinical interviews remain under-researched. In this proof-of-concept study, 10 LLMs assessed transcripts of three simulated psychiatric interviews across all 100 items of the Association for Methodology and Documentation in Psychiatry (AMDP) system, benchmarked against 108 early-career clinicians rating full audiovisual recordings, using an expert consensus panel as reference. GPT-5.1 and Gemini-3-Pro-Preview achieved the highest accuracy (0.72; 64th percentile of the clinician distribution) using majority voting across three runs with AMDP definitions as context. GPT-5.1, selected for a marginal advantage, showed per-scenario accuracies of 0.81 (depression), 0.76 (mania), and 0.60 (schizophrenia) versus clinician means of 0.79, 0.68, and 0.58. Clinicians and LLMs showed distinct error profiles: clinicians tended to over-infer symptom presence, whereas LLMs more conservatively flagged items as “not assessable” — most pronounced for observation-dependent items but present even for text-assessable items (19.4% vs. 11.4%, *p* < 0.001). In post hoc simulated disagreement resolutions (2091 clinician pairs; 35.5% disagreements), LLM and board-certified supervision were associated with more accurate resolutions than unsupervised random clinician selection (*p* < 0.0002). These proof-of-concept findings require validation in real patient interviews, larger samples, and prospective studies integrating multimodal input.

## Introduction

Psychiatry is unique among medical specialties in that its core diagnostic method depends largely on symptoms expressed by spoken language^1–4^. Large language models (LLMs) may therefore transform psychiatric assessment as profoundly as convolutional neural networks transformed radiology^5^. Radiology uses medical imaging to prepare a report that informs diagnosis; psychiatry uses language expressed during the psychiatric clinical interview (pCI) to derive a psychopathological assessment (PPA) that serves the same function. Both are essential intermediate steps between raw data and clinical decisions. In the absence of biomarkers^6^, the pCI has been the cornerstone of psychiatric practice, and the resulting PPA largely determines a patient’s initial clinical trajectory^7^: hospitalization, discharge, referral, and treatment.

This central role of language in psychiatry has stimulated a substantial body of research in the field^8^. Early work used hand-crafted linguistic features and classical machine learning to detect depression or suicidality from texts^9^, demonstrating that meaningful signals could be extracted from language^10,11^. More recently, LLMs^12^ have shown high accuracy on psychiatric tasks^13–15^, including diagnostic classification^10^, questionnaire scoring^16^, and symptom detection from social media posts^9,17,18^, case vignettes^19,20^, and electronic health records^21–24^. However, existing applications skip directly from texts to diagnoses, or use inputs that are not comparable to the pCI, and bypass the intermediate step that defines clinical practice^25^ - a systematic PPA based on the pCI. Embedding-based approaches that compute similarity between free text and symptom descriptors move closer to naturalistic input but still assess only narrow symptom ranges^3,8^. Although some existing studies include clinician comparisons^26–31^, these typically evaluate coarse-grained endpoints such as diagnosis or screening scores rather than structured PPAs, use inputs not comparable to the pCI, and do not characterize clinician error patterns at the level of PPAs. While LLMs hold clear potential for medicine and psychiatry, they are being rapidly and uncritically adopted across clinical settings^32,33^. It therefore remains both unclear and important to investigate whether current LLMs can reliably extract comprehensive PPAs from pCI-transcripts, and to define frameworks for their evaluation.

To address this, we first used clinically representative input: transcripts and videos of representative pCIs (depression, mania, schizophrenia) enacted by a trained simulation patient (SP)^34–36^. Second, we employed an operationalized high-resolution assessment through the Association for Methodology and Documentation in Psychiatry System’s (AMDP) framework^37–39^, using a reduced three-level rating scheme (absent, present, not assessable) as recommended for diagnostic practice. Third, we established a reference rating via expert consensus^40,41^, that serves as the reference standard for both clinician and LLM accuracy. Fourth, we benchmarked LLMs against practicing clinicians from across three German hospitals, providing a real-world comparison with the inter-rater variability that characterizes the PPA in clinical practice^42,43^.

10 LLMs were evaluated for this task using interview transcripts, while clinicians rated the full audio-visual video recordings. This design intentionally compares transcript-only LLM assessment against the clinical standard of audio-visual assessment, as transcript-based evaluation constitutes the most feasible near-term pathway for automated PPAs^44^. Differences in error patterns between clinicians and models were investigated^45,46^ and post-hoc analyses were performed to simulate LLM supervision in disagreement scenarios, compared with supervision by board-certified clinicians (BC)^47,48^.

This study provides proof-of-concept evidence by benchmarking LLMs against practicing clinicians and an expert panel. While generalizability cannot be established from three scenarios, the study enables the first direct comparison of LLM and clinician performance in PPAs from the pCI.

## Results

### Clinician characteristics

The study included 108 unique clinicians (Table 1) with a mean age of 35.3 years and a mean psychiatric experience of 5.4 years (median = 3.0, mode = 1.0). The majority were early-career psychiatry residents (71.3%) and psychologists (11.1%). 32.4% reported German as a non-native language. All participants used AMDP routinely in clinical practice; 72.4% used it as their preferred assessment tool. 17.6% of the raters were AMDP-certified. Each clinician rated between one and three videos depending on which training sessions they attended (90 rated one video, 12 rated two, and 6 rated all three), resulting in 39–53 ratings per video.

**Table 1:** Characteristics of participating clinicians

Trait	Clinicians (*N* = 108)
***Sociodemographic characteristics***	
Age, years, mean (SD)	35.3 (9.4)
No response (*N* (%))	7 (6.5)
**Sex,** *N* **(%)**	
Male	55 (50.9)
Female	48 (44.4)
No response	5 (4.6)
**German as Native Language,** *N* **(%)**	
Yes	55 (50.9)
No	35 (32.4)
No response	18 (16.7)
***Professional role and care setting***	
Years in psychiatry, mean (SD)	5.4 (7.9)
No response (*N* (%))	5 (4.6)
**Primary professional activity,** *N* **(%)**	
Physician in postgraduate training	77 (71.3)
Psychologist	12 (11.1)
Board-certified psychiatrist	7 (6.5)
Senior physician	4 (3.7)
Licensed psychological psychotherapist	2 (1.9)
Head of department/chief physician	2 (1.9)
Lead psychologist	1 (0.9)
No response	3 (2.8)
***Clinical practice and evaluation of psychopathological instruments***	
Psychopathological assessments per month, mean (SD)	36.3 (32.3)
No response (*N* (%))	6 (5.6)
**Last AMDP certification,** *N* **(%)**	
No certification	89 (82.4)
Less than 1 year	6 (5.6)
More than 5 years	3 (2.8)
3–4 years	2 (1.9)
2–3 years	1 (0.9)
1–2 years	1 (0.9)
4–5 years	1 (0.9)
No response	5 (4.6)

*AI* artificial intelligence, *AMDP* Association for Methodology and Documentation in Psychiatry; Full Table in Supplementary Table 12.

### Video quality assessment

All three videos received high ratings across dimensions. The depression video scored highest (overall impression: mean 8.5, SD 1.6, range 2.0–10.0), followed by mania (mean 8.4, SD 1.2, range 4.0–10.0). The schizophrenia video received lower but still favourable ratings (overall impression: mean 7.7, SD 1.9, range 4.0–10.0), with the largest difference on authenticity (7.1 vs. 8.2 and 7.9 for depression and mania, respectively). The overall mean realism rating was 8.2 (SD 1.7) across all three videos (Supplementary Table 1).

### Clinicians and LLMs performance

LLMs were evaluated under two conditions: without AMDP definitions in the prompt (No-K) and with AMDP definitions provided iteratively as in-context knowledge (With-K). Table 2 presents LLM (No-K vs. With-K) and clinician accuracy on AMDP item ratings across the three videos. Clinicians showed video-dependent performance and considerable inter-rater variability (Supplementary Table 2). The overall pairwise percent agreement among clinicians was 65.4% (Supplementary Table 3), with highest accuracy for depression (0.79, SD = 0.10, range: 0.36–0.89, 95% CI [bootstrap, 10,000 iterations]: 0.75–0.82), followed by mania (0.68, SD = 0.09, range: 0.37–0.83, 95% CI: 0.65–0.70), and lowest for schizophrenia (0.58, SD = 0.11, range: 0.36–0.79, 95% CI: 0.54–0.61). The mean accuracy of this predominantly early-career sample across videos was 0.68 (SD = 0.13, range 0.36–0.89, 95% CI 0.65–0.70).

**Table 2:** LLM and clinician accuracy on AMDP ratings across clinical scenarios: Without and with AMDP-definitions

Model	Mania No-K/With-K	Depression No-K/With-K	Schizophrenia No-K/With-K	Overall No-K/With-K
Clinicians	0.68 (0.37 - 0.83)	0.79 (0.36 - 0.89)	0.58 (0.36 - 0.79)	0.68 (0.36 - 0.89)
GPT-5.1	0.72/**0.76**	0.83/0.81	0.55/**0.60**	0.70/**0.72**
Gemini-3-Pro-Preview	0.68/0.72	0.87/**0.87**	0.56/0.58	0.70/**0.72**
Kimi-K2-Thinking	0.70/0.69	0.85/0.77	0.46/0.64	0.67/0.70
Gemini-2.5-flash	0.71/0.67	0.85/0.77	0.54/0.52	0.70/0.65
Claude-Sonnet-4-5	0.66/0.69	0.84/0.68	0.52/0.49	0.67/0.62
Mistral-large-3	0.71/0.69	0.82/0.74	0.39/0.43	0.64/0.62
Qwen3-Next-80B-A3B-Thinking	0.67/0.53	0.84/0.58	0.46/0.48	0.66/0.53
GPT-4o-mini	0.67/0.61	0.83/0.67	0.35/0.34	0.62/0.54
GPT-OSS-20B	0.58/0.57	0.25/0.55	0.38/0.49	0.40/0.54
DeepSeek-R1	0.66/0.53	0.34/0.54	0.55/0.39	0.52/0.49
**Mean** **±** **SD**	0.68 ± 0.04/0.65 ± 0.08	0.73 ± 0.23/.0.70 ± 0.11	0.48 ± 0.08/ 0.50 ± 0.09	0.63 ± 0.10/ 0.61 ± 0.08

With-K = AMDP definitions provided iteratively in the prompt (10 definitions per inference, covering the full transcript across 10 inferences); No-K = no definitions provided. Values represent accuracy on the reduced AMDP scale. For LLMs, each cell shows No-K/With-K accuracy regarding a majority vote across three independent inferences with T0.5. For clinicians, values are mean accuracy with minimum–maximum range across raters in parentheses. Per-video performance under all configurations and non-reduced-scale results are reported in Supplementary Table 6.

The clinician sample included non-native German speakers; however, language background did not significantly predict accuracy after controlling for age, years in psychiatry, certification, and completed AMDP training (β = 0.051, *p* = 0.060; Supplementary Table 4).

Using text transcripts as input while clinicians had access to full audiovisual recordings, most LLMs followed the same video-dependent pattern. The mean accuracy across all models (No-K) was highest for depression (0.73 ± 0.23), intermediate for mania (0.68 ± 0.04), and lowest for schizophrenia (0.48 ± 0.08). GPT-5.1 and Gemini-3-Pro-Preview showed the highest overall performance both for No-K (0.70) and With-K (0.72), marginally exceeding the clinician mean (0.68).

Middle-tier models, including Kimi-K2-Thinking, Gemini-2.5-Flash, and Claude-Sonnet-4.5, recorded overall No-K accuracies between 0.67 and 0.70. The lowest overall No-K accuracies were observed in GPT-OSS-20B (0.40) and DeepSeek-R1 (0.52).

The inclusion of AMDP-Definitions resulted in a marginal numerical decrease of the overall mean accuracy across models, shifting from 0.63 (No-K) to 0.61 (With-K). Scenario-specific mean accuracies also showed marginal changes in means (Schizophrenia: 0.48 to 0.50; Depression: 0.73 to 0.70; Mania: 0.68 to 0.65). For individual models, GPT-5.1 and Gemini-3-Pro-Preview showed a minor increase from 0.70 to 0.72 With-K.

Sensitivity analyses regarding the temperature (T0.0, deterministic; T0.5, moderate sampling variability, Supplementary Table 5) revealed that GPT-5.1 performed comparably best at T0 With-K (0.73) (Supplementary Table 6). The performance of some models, particularly DeepSeek, showed high variability depending on the specific combination of temperature and knowledge settings. Full non-reduced-rating results and further sensitivity experiments regarding temperature, and the detailed usage of knowledge and language settings for selected representative LLMs can be found in Supplementary Table 6 and Supplementary Fig. 1-2.

### LLM vs. Clinicians

During the model selection phase, Gemini-3-Pro-Preview and GPT-5.1 emerged as the most accurate models (With-K). Because GPT-5.1 demonstrated a slight numerical advantage in two of the three clinical scenarios, we selected it for the primary analysis, subsequently referring to it as GPT-5.1-MV, with MV denoting majority voting across three runs with T0.5.

**Fig. 1:**
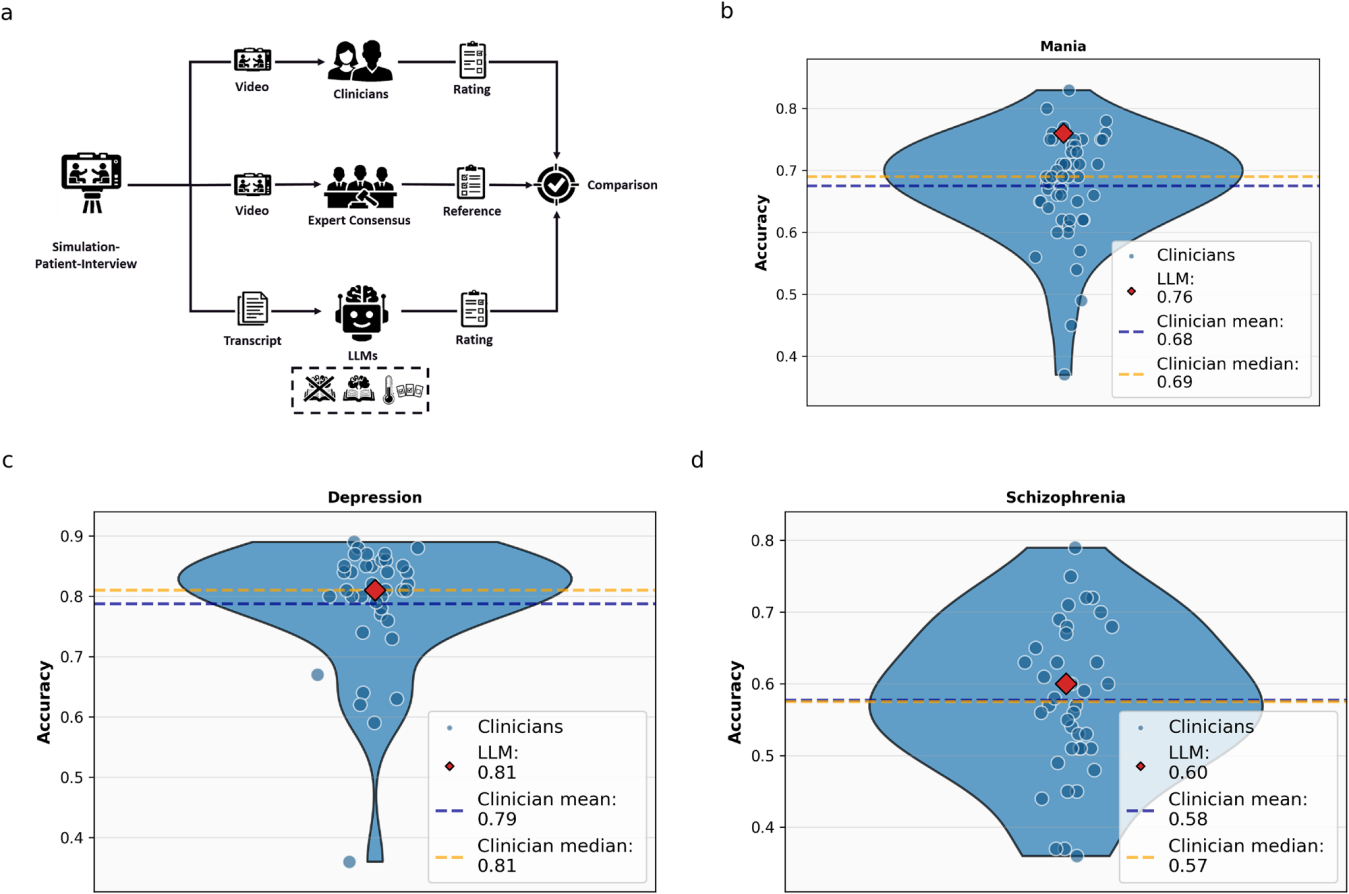
Comparison of clinician and LLM for clinical interviews. **a** Experimental design. Simulated patient interviews were assessed via three pathways: practicing clinicians (*n* = 108), an expert consensus panel (*n* = 3 AMDP trainers establishing ground truth), and LLMs (*n* = 10 models). LLMs evaluated transcripts under two conditions: with/without AMDP definitions (10 definitions per run). Models were queried in JSON mode to ensure structured output. Evaluated models: GPT-5.1, GPT-4o-mini, Claude-Sonnet-4.5, Gemini-3-Pro-Preview, Gemini-2.5-Flash, Mistral-Large-3, Qwen3-Next-80B, DeepSeek-R1, Kimi-K2-Thinking, and GPT-OSS-20B. (b–d) Violin plots showing clinician reduced-accuracy distributions (blue) with the best-performing LLM (GPT-5.1-MV) with majority voting over three runs and knowledge context of 10 AMDP definitions (red diamond) for mania (**b**), depression (**c**), and schizophrenia (**d**). Dashed lines indicate clinician mean (purple) and median (orange). LLM accuracy: mania 0.76 (93rd percentile), depression 0.81 (54th percentile), schizophrenia 0.60 (62nd percentile).

Figure 1 shows the distribution of clinician reduced-accuracy (using full audio-visual input) alongside GPT-5.1-MV performance (using transcript-only input) for each diagnostic category. The model showed slightly better accuracies than the predominantly early-career clinician mean for depression (0.81 vs. 0.79, simulation-derived *p* = 0.62), mania (0.76 vs. 0.68, *p* = 0.03), and schizophrenia (0.60 vs. 0.58, *p* = 0.68), with none of the differences reaching statistical significance after Bonferroni correction for multiple testing (*p* < 0.017). GPT-5.1-MV achieved a total accuracy of 0.72, placing it at the 64th percentile of the distribution of predominantly early-career clinicians (Supplementary Fig. 3).

### LLM errors vs. Clinician errors

Comparing item-level error patterns between GPT-5.1-MV and clinicians across scenarios (100 items × 3), we applied the threshold-based difficulty classification (>70% clinician error rate = difficult; <30% = easy) and identified 12 items with clinician-specific difficulty, 23 with LLM-specific difficulty, and 30 with shared difficulty (Supplementary Fig. 4 and Supplementary Fig. 5). These classifications were robust to clinician exclusion thresholds, with overall classification agreement remaining above 90% across all thresholds (97.0% at 10%, 93.0% at 20%, 90.7% at 30%, 90.0% at 40%; Supplementary Table 7).

To characterize the full error profile of clinicians, we examined all items where clinicians’ modal predictions disagreed with the reference. By this criterion, clinicians committed 69 errors (mania: 23, depression: 11, schizophrenia: 35) and GPT-5.1-MV committed 84 errors (mania: 24, depression: 20, schizophrenia: 40), with both showing the highest error rates for schizophrenia. Error patterns differed by reference rating category: clinician errors were dominated by misclassifying “not assessable” items as “absent” or “present” (44 of 69 errors), whereas GPT-5.1-MV errors were concentrated on rating “absent” items as “not assessable” (28 of 84 errors). False positive and false negative counts were comparable (14 vs. 9 and 6 vs. 5, respectively) (Table 3 and Supplementary Fig. 6).

**Table 3:** Distribution of rating errors by direction for GPT-5.1-MV - and clinicians across clinical scenarios

Error direction	Mania		Depression		Schizophrenia	
	*LLM*	*Clinician*	*LLM*	*Clinician*	*LLM*	*Clinician*
Absent → Present	3	2	2	4	4	8
Absent → Not Assessable	8	0	9	0	11	3
Present → Absent	2	4	2	1	1	1
Present → Not Assessable	1	0	4	0	4	2
Not Assessable → Absent	8	13	3	6	12	9
Not Assessable → Present	2	4	0	0	8	12
**Total errors**	**24**	**23**	**20**	**11**	**40**	**35**

Errors are defined as disagreements with expert consensus ratings on the reduced AMDP scale (absent, present, not assessable). Clinician errors reflect items where the modal clinician rating disagreed with the expert consensus. LLM results reflect GPT-5.1 with majority voting across three independent runs with AMDP definitions (GPT-5.1-MV).

Of the 100 AMDP items, 9 were classified as observation-dependent (OD) based on the AMDP definitions (perplexity, blunted affect, affective lability, affective incontinence, affective rigidity, motor restlessness, parakinesia, mannerisms, histrionics). On these items, GPT-5.1-MV marked 17 of 27 OD item–video pairs (63.0%) as “not assessable”, whereas the clinician consensus (modal rating) never rated any OD item as not assessable (0.0%; McNemar’s exact test, *p* < 0.001), and even individual clinicians rarely did (3.9%). Even among non-OD-items, the LLM used “not assessable” slightly more frequently than the clinician consensus (19.4% vs. 11.4%; McNemar’s χ² = 11.03, *p* < 0.001; Supplementary Table 8). In the schizophrenia scenario, none of the nine OD items were correctly assessed by the LLM (Supplementary Table 9). When OD items were excluded, LLM accuracy increased from 0.60 to 0.66 for schizophrenia, from 0.76 to 0.81 for mania, and from 0.81 to 0.85 for depression, whereas clinician accuracy relatively decreased due to the exclusion of OD items (Supplementary Table 10).

For items rated as absent by the expert consensus, GPT-5.1-MV showed higher accuracy than the clinician mean across all three scenarios especially when OD items were excluded (depression: 0.86 vs. 0.82; mania: 0.88 vs. 0.80; schizophrenia: 0.76 vs. 0.64; Supplementary Fig. 7).

**Fig. 2:**
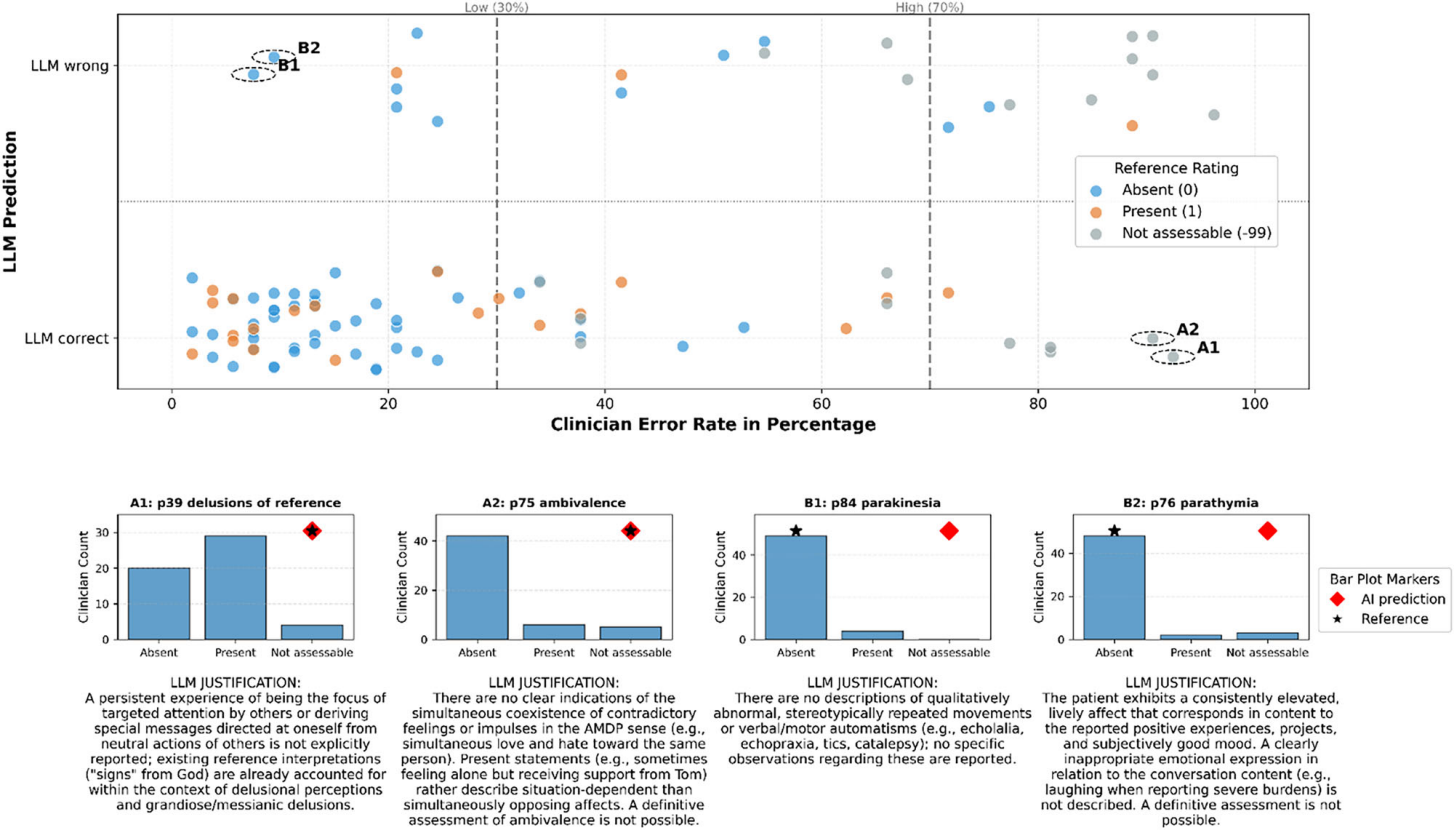
Item-level divergence between Clinician error rates and GPT-5.1 majority voting (Mania). Scatterplot of AMDP items by clinician error rate (x-axis; percentage error of clinicians) and GPT-5.1-MV prediction accuracy (y-axis; majority voting). Points are colored by expert reference rating: absent (blue), present (orange), or not assessable (gray). Vertical dashed lines demarcate low (≤30%) and high (≥70%) clinician error zones. Four example items illustrate error patterns: A1–A2 (bottom right) show LLM-correct items with high clinician error; B1–B2 (top left) show clinician-correct items where the LLM erred. (Bottom) Detailed breakdown for each example item showing clinician rating distributions (bars), LLM prediction (red diamond), expert reference (star), and LLM justification text. An interactive visualization enabling inspection of all items with LLM rationales is available in the online repository Mania, Depression, Schizophrenia.

Figure 2 illustrates the distribution of items by clinician error rate alongside GPT-5.1-MV ratings and rationales for the mania scenario. Interactive versions for all three scenarios are available online Mania, Depression, Schizophrenia.

### Investigating potential synergistic effects

**Fig. 3:**
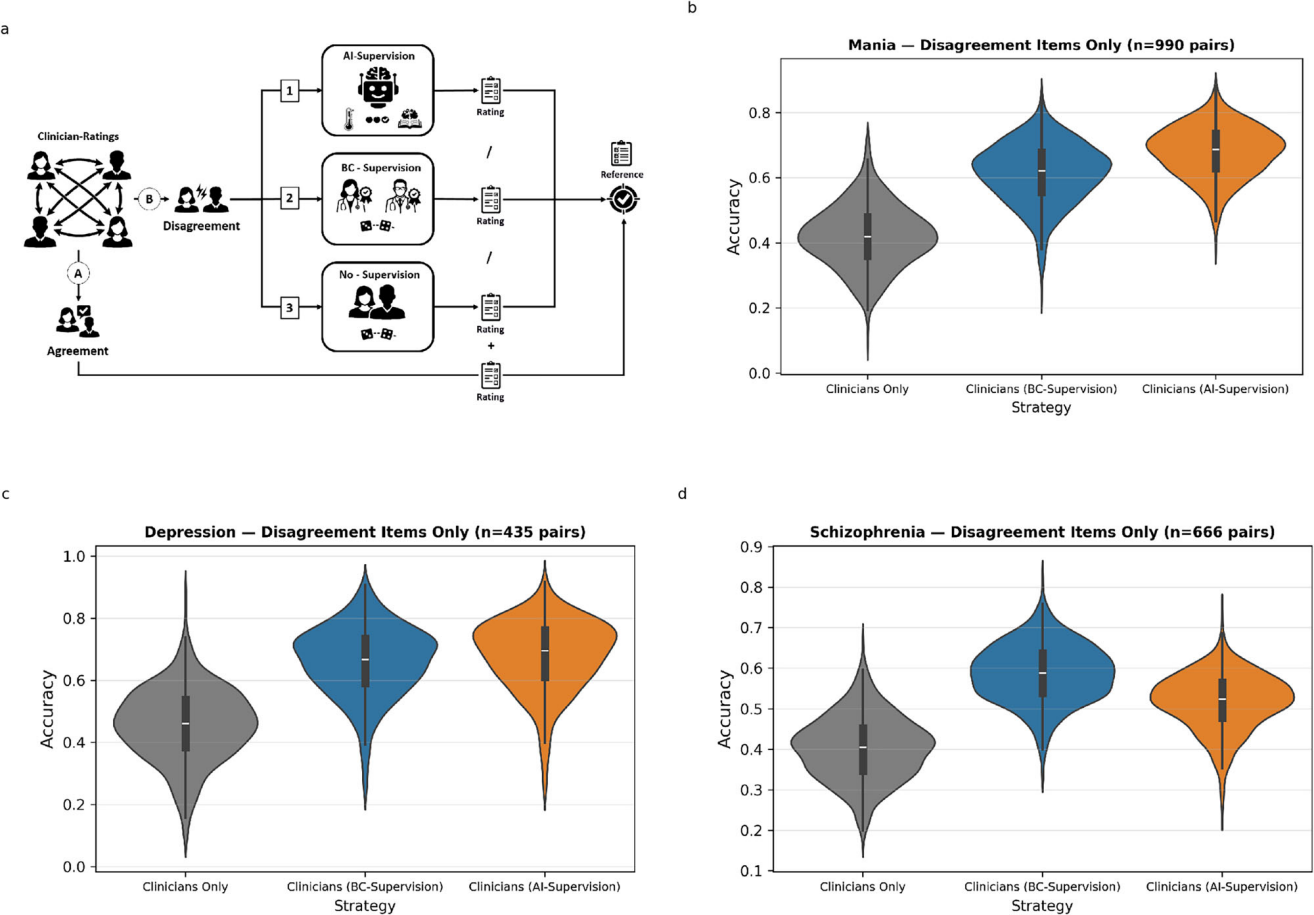
Clinician LLM supervision simulation framework. **a** Post-Hoc Simulation design for evaluating AI-supervised psychiatric assessment. From a pool of 108 clinician ratings, pairs were randomly sampled to build clinical teams (for all videos, 2,091 pairs). Path A (Agreement): When both clinicians agreed on a symptom rating, their consensus was adopted. Path B (Disagreement): When clinicians disagreed, three supervision strategies were compared: (1) AI Supervision: The best-performing LLM (GPT-5.1-MV) provided the deciding rating. (2) Board-Certified Supervision: A randomly selected rating from the pool of board-certified psychiatrists who independently assessed the same video resolved the disagreement. (3) Random Selection: Random selection between the two disagreeing clinicians (baseline control; clinicians only). All three strategies were evaluated against the expert consensus ground truth. The combined ratings from agreement cases (Path A) and disagreement resolutions (Path B) were aggregated to calculate overall accuracy for each supervision approach. Violin plots showing accuracy distributions across simulated team configurations for mania (**b**), depression (**c**), and schizophrenia (**d**) for disagreement items.

Since the error patterns of the predominantly early career clinicians and GPT-5.1-MV were partially non-overlapping, we explored whether GPT-5.1-MV supervision showed signs of improving clinical decision-making (Fig. 3). We simulated collaborative scenarios using 2091 pairs of clinician raters across the three scenarios. When clinicians disagreed on an item rating (35.5% of cases), we compared three resolution strategies (Fig. 3a): Clinicians Only - Random Choice between disagreeing clinicians, Clinicians with BC Supervision (mania: *n* = 8, depression: *n* = 9, schizophrenia: *n* = 3), and Clinicians with LLM-Supervision; in each case, the chosen rating replaced the disagreement and was used as the final rating for that item. Random selection among disagreeing clinicians yielded accuracies of 0.42 (depression), 0.46 (mania), and 0.41 (schizophrenia). Both LLM and BC supervision significantly (*p* < 0.0002, permutation test) outperformed this baseline across all three scenarios (Table 4). The LLM showed the largest advantage in the depression video (Δ = 0.27 vs. 0.21) and mania video (Δ = 0.25 vs. 0.22), while BC supervision performed slightly better in the schizophrenia video (Δ = 0.18 vs. 0.11).

**Table 4:** Supervision strategy simulation outcomes for the permutation test

Scenario	Random selection	BC supervision	LLM supervision	Δ BC vs random	Δ LLM vs random
Depression	0.42	0.63	0.69	0.21	0.27
Mania	0.46	0.68	0.71	0.22	0.25
Schizophrenia	0.41	0.59	0.52	0.18	0.11

All *p* < 0.0002, two-sided (permutation test, 5000 permutations). Accuracies reflect performance on items where clinician pairs disagreed (35.5% of ratings). *BC* board-certified clinician, *LLM* Large Language Model.

## Discussion

In this proof-of-concept study, we evaluated 10 LLMs on PPAs from three simulated pCIs and benchmarked their performance on corresponding transcripts against 108 practicing, predominantly early-career clinicians (71.3% residents), who were provided video and audio, using the AMDP system. High realism ratings across all three scenarios suggest that the simulated interviews provided a sufficiently realistic approximation of clinical encounters for this study, consistent with prior literature on the use of SP^34–36^.

Our key findings were: (1) GPT-5.1 showed the highest accuracy among tested models, and the primary-comparison configuration (GPT-5.1-MV: 0.72) performed approximately at the mean level within the clinician distribution; (2) clinicians and GPT-5.1-MV exhibited diverging, partially complementary error patterns; and (3) in post-hoc simulated disagreement resolution, both LLM-(GPT-5.1-MV) and BC-supervision were associated with significantly higher accuracy than random selection.

Performance varied across model families, with proprietary models yielding higher accuracies on the tasks than open-weight alternatives^28,49,50^. GPT-5.1 and Gemini-3-Pro-Preview showed the highest overall accuracy across knowledge-configurations (No-K: 0.70/With- K: 0.72). Interestingly, providing AMDP definitions did not uniformly improve performance across models for the given three scenarios. The overall mean accuracy slightly decreased from 0.63 to 0.61 when definitions were included. While the Schizophrenia scenario saw a marginal average improvement (0.48 to 0.50), accuracies for Depression and Mania were lower overall. However, the highly parametrized models GPT-5.1 and Gemini-3-Pro-Preview showed no decrease, but rather a slight numeric increase in overall performance. This resilience may be due to their larger parameter scale affording superior context integration and attention allocation. This heterogeneity could reflect known model-specific nuances in handling injected context, including the “lost in the middle” phenomenon in long contexts^51^, conflicts between external definitions and pretrained priors^52^, and model-specific variation in instruction-following^53^, but could also be attributed to noise.

Temperature sensitivity analyses (T0.0 vs. T0.5) suggested a marginal performance difference for GPT-5.1 With-K (Δ=+1). Temperature effects were marginal and inconsistent across transcripts and models.

We emphasize that a definitive ranking cannot be established due to the small number of scenarios and the minor accuracy differences. However, the observation that the best-performing models on our task also ranked highest on general capability benchmarks at the time of the study, and that GPT-5.1 and Gemini-3-Pro-Preview maintained the highest accuracy across sensitivity analyses, suggests that the results are unlikely to be entirely attributable to chance. At the same time, evaluating multiple models across configurations and selecting the best-performing combination for the primary comparison introduces a winner’s curse that inflates performance estimates. Accordingly, these findings should be interpreted as exploratory upper bounds.

GPT-5.1-MV performed approximately at the mean level of a distribution of a clinician sample, being predominantly in early career stages in all three scenarios. In line with previous studies, a high variability in the accuracy of clinician ratings was observed in our analysis (overall accuracy = 0.36–0.89)^42,43^. The percentile ranks and bootstrap confidence intervals for the clinician mean provide a more robust characterization of the LLM’s position within the clinician distribution. A similar performance pattern of LLMs has been observed across medicine^27,30^: A meta-analysis of 83 studies reported an overall generative AI diagnostic accuracy of 52.1%, comparable to non-expert but significantly below expert physicians^29^. In psychiatry specifically, LLMs matched or exceeded clinician panels on diagnostic classification of schizophrenia spectrum disorders^26^ and Obsessive-Compulsive Disorder^19^. The performance of LLMs relative to experienced, board-certified psychiatrists could not be reliably inferred, as the number of clinicians in this career stage was very low in the current sample (e.g., schizophrenia *n* = 3). A clinician sample with a higher proportion of experienced AMDP specialists would likely increase the accuracy.

Beyond overall accuracy, the error profiles of clinicians and GPT-5.1-MV diverged systematically. Clinician errors were dominated by misclassifying “not assessable” items as “absent” or “present”, reflecting a tendency to infer symptom status when interview information is insufficient^54,55^. GPT-5.1-MV errors followed the opposite pattern: items that were rated as “absent” were frequently classified as “not assessable” by the models. Based on qualitative analyses of the reasoning-outputs of the model, we hypothesized that this discrepancy was primarily driven by the absence of visual input in the transcript-only condition (e.g., parakinesia: “no specific observations regarding these are reported”, Fig. 2). The input asymmetry was confirmed by the OD item analysis: GPT-5.1-MV rated 63.0% of OD items as “not assessable”, compared to 0.0% in the clinician consensus and 3.9% among individual clinicians. The effect was most pronounced in the schizophrenia scenario, where GPT-5.1-MV failed on all nine OD items, indicating that the transcript-only input limitation disproportionately affected this diagnosis. For this category of symptoms where visual input can be crucial for the diagnosis (e.g., parakinesia for catatonic schizophrenia), transcript-only assessment is insufficient in real-world clinical settings, even when aggregate accuracy appears adequate. Visual input could potentially improve performance (for schizophrenia, from 0.60 to 0.66 when OD items are excluded), but it remains uncertain whether current multimodal LLMs can reliably interpret nonverbal clinical cues and close this gap even when visual input is available^44^.

However, the difficulty of the schizophrenia scenario cannot be attributed to the transcript-only limitation alone: clinicians, with full audiovisual input, also showed the highest inter-rater variability and lowest performance for this diagnosis, consistent with prior literature reporting lower reliability for schizophrenia^43^. Several factors may have contributed to this shared difficulty: the schizophrenia video received lower authenticity ratings (7.1 vs. 8.2 and 7.9 for depression and mania), which may have made assessment more difficult for both groups, and schizophrenia involves a broader range of psychopathological domains, increasing the number of items requiring nuanced clinical judgement. The influence of the SP was minimized by using the same trained SP for all three videos. Potential interviewer effects were standardized by using exclusively AMDP-trained psychiatrists as interviewers.

The tendency to rate items as “not assessable” did not seem to be fully explained by missing visual information or the schizophrenia scenario: even for items not classified as OD, the GPT-5.1-MV used this category more frequently than the clinician consensus (19.4% vs. 11.4%, *p* < 0.001). This may suggest that GPT-5.1-MV tends toward a stricter evidentiary standard for inferring symptom assessability. Consistent with this interpretation, when clinicians misclassified expert-confirmed absent items, the dominant error was over-inference of symptom presence (14 of 17 errors, 82%; Table 3 and Supplementary Fig. 6). GPT-5.1-MV showed the opposite pattern: when it erred on absent items, the majority were conservative misclassifications as ‘not assessable’ (28 of 37 errors, 76%), with fewer false positives (9 of 37, 24%). This divergence was reflected in overall “absent item” accuracy, for which GPT-5.1-MV performed better than clinicians across all three scenarios (Supplementary Fig. 7), especially when OD items were excluded from the analyses.

Based on these findings we hypothesized that clinicians and LLMs may employ complementary interpretive strategies: clinicians draw on contextual and nonverbal cues — aided by full audiovisual input — but may over-infer from incomplete information, while LLMs adhere more strictly to available textual evidence and operationalized definitions.

This complementarity motivated the post-hoc experiment of combining GPT-5.1-MV and clinician ratings in a disagreement scenario, testing whether their diverging, partially complementary error profiles could be leveraged to improve overall assessment accuracy. Post-hoc simulated LLM-supervision by GPT-5.1-MV or BC was significantly associated with higher accuracy compared with the random decisions baseline when raters disagreed. The pattern of improvement appeared to differ by clinical presentation, with LLM supervision showing the largest gains in depression and mania scenarios, and BC supervision in schizophrenia. The lower effectiveness of LLM supervision in the schizophrenia scenario likely reflects the same transcript-only limitations discussed above. These findings are exploratory, based on simulation, and cannot inform clinical implementation. In particular, the low BC-count — likely due to limited availability during recruitment — can bias toward higher or lower performance regarding BC-supervision. Nonetheless, the consistency of the effect across all three scenarios and both supervision conditions suggests that LLM-based decision support in situations of clinical uncertainty emerges as a strong hypothesis warranting prospective validation^45,47,48^.

Several strengths and limitations should be considered in interpreting these findings. The focus on three interviews with dense annotation and participation of multiple early-career practicing clinicians enabled detailed item-level and rater-level analyses. The use of SPs ensured standardized input, addressed ethical and data protection constraints, enabled open release with benchmarking and enhanced transparency.

The small number of interviews limits the scope of inferential claims and precludes any generalizability. Any inferential claim regarding LLM-versus-clinician performance or disagreement-resolution rests on resampling and simulation within three interviews and should be interpreted with caution, as the resulting confidence intervals quantify uncertainty only for these specific videos under the specific rating setup used in this study. A small number of clinicians rated more than one video, which may introduce non-independence across videos, though the majority (83%) rated only one. Despite their realism, simulated encounters cannot fully capture the complexity of real clinical interactions. The single-video-per-diagnosis design does not allow diagnosis-level differences in accuracy to be attributed to specific interview, rater, or model characteristics. German-language focus may also further limit generalizability. Additionally, testing multiple LLM families across different configurations on the same dataset increases the chance of selecting a favourable model, overestimating accuracy. Furthermore, the clinicians of the sample were predominantly in their early careers. LLM performance relative to experienced, AMDP-certified psychiatrists may differ substantially.

The OD item analysis highlights that the differences in input type cannot be dismissed. Clinicians received full audiovisual input and LLMs processed only transcripts, which does not make the task interchangeable. While this asymmetry is inherently limiting, it was an intentional and necessary design choice: current vision-language models lack the temporal integration^44^ and nonverbal cue interpretation required for psychiatric assessment^56,57^, making transcript-based evaluation the most plausible near-term pathway. At the same time, providing clinicians with full audiovisual recordings rather than transcripts ensured the most realistic assessment conditions, which was essential for engagement with the task.

In summary, in this proof-of-concept study, we evaluated 10 LLMs from both proprietary and open-source model families on transcript-based psychopathological assessments derived from three simulated pCIs, benchmarking their performance against 108 predominantly early-career practicing clinicians who assessed the full audiovisual recordings using the AMDP system. GPT-5.1-MV and its Gemini counterpart achieved the highest overall accuracy. Performing approximately at the mean level within the clinician distribution, GPT-5.1-MV exhibited distinct error profiles that appeared partially complementary to clinician errors. Exploratory analyses of simulated disagreement resolution suggest that LLM supervision may improve accuracy in cases of clinical uncertainty. Embracing open-science practices, we enable researchers to benchmark current and future models and inform their own deployment decisions.

Key next steps include validation on real patient interviews, acknowledging the ethical and data protection challenges this poses for certain diagnoses, as well as extension to other languages and prospective studies embedding LLM supervision into clinical workflows to test whether post-hoc accuracy gains translate to real-world decision-making. As multimodal models mature, future evaluations should reassess whether direct video-based assessment can address the OD items that currently limit transcript-only approaches.

## Methods

### Clinical interviews

Three semi-structured clinical interviews (24-33 minutes) were conducted with a SP portraying psychopathological syndromes typical for depression, mania, and schizophrenia. We chose simulated interviews to: **1**. enable submission of transcripts to commercial APIs, which would be prohibited under current regulations, **2**. allow open-source release for benchmarking, and **3**. guarantee content was absent from LLM training corpora^58,59^. This aligns with psychiatric education standards, where SPs yield judgements comparable to real encounters^34–36^. AMDP-trained psychiatrists conducted the PCIs with the SP to maintain naturalistic dynamics (Supplementary Fig. 8). The trained SP received approximately one hour of clinical briefing from a clinician regarding the specific disorder and relevant item definitions for each video, but performed without a script. The interviews unfolded naturally, with the simulation patient improvising responses based on their understanding of the clinical presentation, preserving the unstructured conversational dynamics typical of real psychiatric encounters.

### Psychopathological assessment instrument

For the PPA, we employed the AMDP^37–39^. It is internationally recognized, translated into multiple languages, and provides a comprehensive terminology of psychopathological symptoms with operationalized definitions^37–39,60,61^.

We used the 11th German version (100 items rated absent to severe and “not assessable” for insufficient information based on the interview)^37^.

AMDP’s advantages include **1**. semi-structured format reflecting real pCIs where symptoms emerge through dialogue rather than fixed questions, **2**. operational definitions with clinical examples enabling validation and serving as a reference knowledge base for LLMs, **3**. widespread usage in German-speaking psychiatry^62^, allowing comparison with clinicians, and **4**. coverage of multiple psychopathologies^39^.

We report results under two rating schemes: the original four-level scheme (0 = absent, 1 = mild, 2 = moderate, 3 = severe, 4 = not assessable), referred to as the non-reduced version, and a simplified three-level scheme (0 = absent, 1 = present, 2 = not assessable), referred to as the reduced version. The reduced version collapses all severity grades into a single “present” category, following the AMDP’s recommendation for diagnostic practice^37^, since major psychiatric classification systems require only the presence of a symptom to fulfil a diagnostic criterion. The reduced version serves as the primary outcome measure for all analyses; non-reduced results are reported in the supplements.

### Ground truth establishment

Expert consensus ratings were established by a panel of three board-certified senior psychiatrists, all contributors and trainers of the current edition of the AMDP manual, who served as the reference standard for all subsequent analyses. To ensure maximum diagnostic precision, panel members initially reviewed each video independently before meeting to achieve a unanimous consensus through discussion. This exhaustive process required approximately three hours per video and was employed to establish a definitive ground-truth benchmark, which is essential in clinical contexts lacking an objective gold standard^40^.

### LLM assessment procedures

Videos were transcribed locally using OpenAI’s Whisper large-v3 model. Speaker diarization was performed to label interviewer and patient turns. No behavioural annotations were included. All transcripts underwent manual review by the main investigator and correction against the original audio to ensure accuracy. These transcripts served as input for the LLMs. Proprietary and open-source LLMs were selected based on their rankings on the LMSYS Chatbot Arena leaderboard (11/2025)^63^. The following models were evaluated: GPT-5.1, GPT-4o-mini, Claude-Sonnet-4.5, Gemini-3-Pro-Preview, Gemini-2.5-Flash, Mistral-Large 3(2512), Qwen3-Next-80B, DeepSeek-R1, Kimi-K2-Thinking, and GPT-OSS-20B (Fig. 1a). Proprietary models were accessed through their respective APIs (OpenAI, Google, Anthropic, and Mistral). Open-source models (including GPT-OSS-20B) were accessed via the Together AI API (Together AI, Inc., San Francisco, CA). Model versions, access dates, inference costs, and runtime are reported in Supplementary Table 11.

A standardized prompt, following the GRASCEF framework (Goal, Role, Action, Steps, Context, Example, Format)^64,65^, was formulated, not iteratively optimized, and remained fixed before data collection to prevent tailoring to the dataset. It assigned the LLM the role of a rule-based clinical diagnostic classifier operating strictly within AMDP criteria. For each inference, the model received the full interview transcript and was instructed to evaluate each AMDP item by searching for indicative phrases, behavioural descriptions, and symptom indicators in the text, assigning a severity rating along with a brief clinical justification based on the AMDP. The model was constrained to base its classification solely on the provided text without interpretation beyond AMDP definitions and to return structured JSON output. The complete prompt is provided in Supplementary Note 1.

The LLM selection phase addressed two screening questions: Whether providing AMDP definitions improves accuracy and which model family performs best.

Models were evaluated under two definition conditions: a zero-knowledge (No-K) condition (no AMDP definitions provided, relying on the internal priors of LLMs) and a full-definition condition, where the same transcript was processed 10 times, each with a different batch of 10 symptom definitions^66^, until all 100 items were evaluated iteratively (With-K).

A majority vote on the reduced scales for three independent inferences per model was applied. This approach was included for its documented ability to mitigate hallucinations and improve reliability in LLM reasoning tasks^67^. The majority vote was limited to three independent inferences as the mathematical minimum required for consensus while maintaining computational feasibility. The “With-K” condition already involved 10 iterative sub-evaluations per transcript. Increasing the number of iterations further was deemed impractical, since it would be overly time-intensive, therefore lacking practical utility in real clinical settings.

Additional post-hoc sensitivity analyses assessed the influence of temperature (0.0, 0.5), language (all prompts and transcripts translated from German to English using GPT-5.1 with subsequent human verification; Supplementary Note 1), and a finer-grained number of AMDP definitions per batch (2, 5, 8, 10). These sensitivity analyses did not inform the selection of the final model or configuration.

### Clinician assessment

Practicing clinicians from three psychiatric hospitals in Germany (a university hospital, a university-affiliated research hospital, and a regional psychiatric care center) were recruited during routine training sessions between April and November 2025. Inclusion criteria were current direct patient contact and routine use of AMDP. Each clinician, independently and anonymously^68^, rated one to three videos depending on availability during the training session. Clinicians had access to the AMDP manual. Clinicians completed paper-based AMDP rating forms during and immediately after viewing, within video duration plus an additional 15 minutes, reflecting time constraints typical of clinical practice^69,70^. Clinicians additionally rated the simulation quality of each video across six dimensions (realism, consistent portrayal, expressiveness, responsiveness, authenticity, and overall impression) on a 1–10 scale. Clinician ratings with more than 80% of items left unrated (*n* = 6) were excluded prior to data digitalization, as these were considered insufficiently complete to represent meaningful assessments. Remaining ratings were visually inspected for signs of disengagement, but no further exclusions were warranted.

### Statistical Analyses

To characterize clinician performance, we calculated the proportion of correctly rated items for each clinician per video, yielding a distribution of clinician accuracy. Bootstrap 95% confidence intervals for the clinician mean were computed by resampling clinicians with replacement (10,000 iterations).

The LLM’s accuracy overall and for each scenario was compared by computing its percentile rank among clinicians.

For our primary analysis, we compared the best-performing LLM (utilizing the With-K configuration and majority voting) against clinicians on the reduced AMDP scale, evaluated separately for each video. To test whether the LLM’s accuracy differed significantly from that of an average clinician, we simulated 50,000 synthetic raters, each responding to all 100 items with success probabilities equal to the observed clinician proportion correct per item; the proportion of simulated raters achieving accuracy equal to or greater than the LLM, multiplied by two, yielded two-sided p-values.

To test whether transcript-only input disproportionately limited the LLM’s ability to assess OD items (perplexity, blunted affect, affective lability, affective incontinence, affective rigidity, motor restlessness, parakinesia, mannerisms, histrionics), we compared “not assessable” rates between the LLM and clinician consensus using McNemar’s test for paired nominal data. Each item × video pair served as a matched observation (OD: *n* = 27; non-OD: *n* = 273), with the binary outcome being whether the rater coded “not assessable” versus providing a rating. The clinician consensus was defined as the modal rating across all clinicians for each pair. The exact binomial variant was used when discordant pairs were fewer than 25.

We analyzed item-level error patterns to identify where clinician and best-performing LLM assessments diverge. We calculated item-wise clinician error rates and cross-tabulated them with LLM accuracy. For this analysis, we focused on extreme cases and classified items into four categories: shared difficulty (clinician error rate >70% and LLM incorrect), LLM-specific difficulty (clinician error rate ≤30% and LLM incorrect), clinician-specific difficulty (clinician error rate >70% and LLM correct), and low difficulty (clinician error rate ≤30% and LLM correct)^71^. To assess whether item-level error patterns were robust to noise introduced by low-performing raters, we performed sensitivity analyses excluding the lowest-performing 10%, 20%, 30%, and 40% of clinicians based on overall accuracy.

We post-hoc simulated disagreement resolution on the item-level using unique clinician pairs who assessed the same video. When clinicians diverged in their answer to a certain AMDP item, we compared three resolution strategies: (1) random selection between the two clinicians (baseline), (2) supervision by BC psychiatrists (including senior physicians and department heads) who rated the same video, and (3) LLM supervision utilizing the best-performing model identified in our preceding analyses (configured with the With-K condition and majority voting across three inferences) (Fig. 3a), where the supervision defined the final rating. To compare strategies, we conducted permutation tests for each video, focusing on items on which the two raters disagreed. For each disagreement, three adjudicated responses were available (random, BC, or LLM). Under the null hypothesis of no strategy effect, we randomly permuted strategy labels within each item-pairing unit while keeping responses, item identity, and pairing fixed. The test statistic was the difference in mean accuracy between strategies (BC vs. random; LLM vs. random). Two-sided p-values represent the proportion of 5000 permutations yielding an absolute test statistic at least as large as the observed value.

This study was approved by the Ethics Committee II of Ruprecht-Karls-Universität Heidelberg, Medical Faculty Mannheim (reference number: 2024-567) in accordance with the Declaration of Helsinki. Written informed consent was obtained from all participating clinicians, expert board members and the simulated patient and interviewer. Written consent to publish identifiable images and video recordings was obtained from the simulated patient and interviewer.

## Supplementary information

eSupplementary_v_2.1.

## Data Availability

The interview transcripts and rating data generated and analysed during the current study are publicly available in the GitHub repository https://github.com/42elenz/LLMs_psychopathologies. These data include the transcripts used for large language model assessment and the rating data from the large language models and participating clinicians. The corresponding audiovisual recordings are available from the corresponding author upon reasonable request.

## Code availability

The code can be found in the GitHub repository https://github.com/42elenz/LLMs_psychopathologies. This includes the evaluation pipeline for querying proprietary LLMs via their respective APIs and open-source models via the Together AI API, as well as an inference pipeline based on vLLM for local GPU deployment of open-source models.
