# Supplementary material for: Benchmarking large language models against practicing clinicians on psychopathological assessment
Source: NPJ Digit Med. 2026 Jul 7;9:518. doi: 10.1038/s41746-026-02852-7 (PMC13342660; doi:10.1038/s41746-026-02852-7)
Supplement: Supplementary file 1 — eSupplementary_v_2.1. [file 41746_2026_2852_MOESM1_ESM.pdf]

Supplements

Contents – Numbered by type and ordered by mention in the manuscript

Supplementary Table 1. Simulation-video quality ratings

Supplementary Table 2. Clinician performance summary across clinical scenarios

Supplementary Table 3. Inter-rater agreement among clinicians on reduced labels

Supplementary Table 4. OLS regression: clinician accuracy predicted by German native language, controlling for confounders

Supplementary Table 5. Temperature accuracy gains per model

Supplementary Table 6. LLM performances per scenario for all models

Supplementary Figure 1. Optimization of knowledge and temperature parameters

Supplementary Figure 2. Comparison of LLM accuracy on AMDP psychiatric ratings using original German versus translated English materials

Supplementary Figure 3. Distribution of clinician raters with Best-Performing-LLM with majority voting across all scenarios

Supplementary Table 7. Sensitivity analysis of item difficulty classification across clinician exclusion

Supplementary Figure 4. Item-level divergence between clinician error rates and GPT-5.1 majority voting

Supplementary Figure 5. Reference rating distribution by difficulty type

Supplementary Figure 6. Misclassification patterns of LLMs and clinicians: reference to predicted

Supplementary Table 8. “Not assessable” ratings for observation-dependent items: LLM vs. clinician consensus

Supplementary Table 9. Correctness of LLM ratings on observation-dependent items across scenarios

Supplementary Table 10. Performance excluding observation-dependent items for LLM and clinicians

Supplementary Figure 7. Accuracy on absent items: LLM vs. clinician performance by observation dependency

Supplementary Figure 8. Example of a video setup for clinician rating

Supplementary Table 11. Cost and runtime information for all models for one comprehensive rating (example: mania)

Supplementary Note 1. Prompts German and English

Supplementary Table 12: Characteristics of participating clinicians - full table

Supplementary Table 1. Simulation-video quality ratings

**Quality Dimension**	**Depression Video**	**Mania Video**	**Schizophrenia Video**	**Overall**
Realism	8.6 (1.7) [2.0-10.0]	8.3 (1.3) [4.0-10.0]	7.5 (1.9) [3.0-10.0]	8.2 (1.7) [2.0-10.0]
Consistent Portrayal	8.6 (1.7) [2.0-10.0]	8.2 (1.4) [5.0-10.0]	8.2 (1.6) [3.0-10.0]	8.3 (1.5) [2.0-10.0]
Expressiveness	8.5 (1.8) [2.0-10.0]	8.2 (1.6) [2.0-10.0]	8.0 (1.5) [5.0-10.0]	8.2 (1.6) [2.0-10.0]
Responsiveness	8.3 (1.8) [2.0-10.0]	8.3 (1.5) [2.0-10.0]	8.4 (1.4) [5.0-10.0]	8.3 (1.5) [2.0-10.0]
Authenticity	8.2 (1.7) [2.0-10.0]	7.9 (1.6) [1.0-10.0]	7.1 (2.0) [4.0-10.0]	7.8 (1.8) [1.0-10.0]
Overall Impression	8.5 (1.6) [2.0-10.0]	8.4 (1.2) [4.0-10.0]	7.7 (1.9) [4.0-10.0]	8.2 (1.6) [2.0-10.0]

Data are presented as mean (standard deviation). Ratings were based on a 10-point scale (1 = very poor; 10 = excellent). Each dimension received 30 to 53 ratings per video. Ranges across all dimensions and videos spanned 1.0 to 10.0.

Supplementary Table 2. Clinician performance summary across clinical scenarios

**Video Input Type**	**Accuracy not reduced**	**SD not reduced**	**Accuracy reduced**	**SD reduced**
Mania	0.60 (0.23 - 0.75)	0.09	0.68 (0.37 - 0.83)	0.09
Depression	0.70 (0.24 - 0.82)	0.11	0.79 (0.36 - 0.89)	0.10
Schizophrenia	0.50 (0.26 - 0.73)	0.11	0.58 (0.36 - 0.79)	0.11
All Scenarios	0.60 (0.23 - 0.82)	0.13	0.68 (0.36 - 0.89)	0.13

SD = standard deviation; Accuracy values represent mean proportion correct of clinicians for reduced and non-reduced ratings against expert consensus, with ranges (minimum–maximum) across individual clinicians in parentheses.

Supplementary Table 3. Inter-rater agreement among clinicians on reduced labels

**Scenario**	**Raters (n)**	**Items (n)**	**Fleiss kappa**	**Agreement (%)**	**Absent (%)**	**Present (%)**	**Not Assessable (%)**
Mania	53	100	0.37	66.3	61.4	28.5	10.1
Depression	39	100	0.47	73.8	65.8	26.1	8
Schizophrenia	40	100	0.31	55.8	43.5	33.6	22.9
Overall	39-53	100	0.39	65.4	57.3	29.3	13.4

Fleiss' κ values should be interpreted alongside percent agreement, as the high proportion of absent ratings across all scenarios inflates expected chance agreement, attenuating κ (the kappa paradox). 90 clinicians rated one scenario, 12 rated two, and 6 rated all three.

Supplementary Table 4. Ordinary least squares regression: clinician accuracy predicted by German native language, controlling for confounders

	**Coef.**	**Std. Err.**	**t**	***p***	**Lower 95% CI**	**Upper 95% CI**
**Intercept**	0.600	0.066	9.10	<0.001	0.469	0.731
**Native German (binary)**	0.051	0.026	1.91	0.060	-0.002	0.103
**Age**	-0.002	0.002	-0.77	0.442	-0.006	0.002
**Years in psychiatry**	0.004	0.003	1.57	0.120	-0.001	0.009
**Certified (binary)**	-0.024	0.046	-0.54	0.593	-0.115	0.066
**Training completed**	0.012	0.045	0.27	0.785	-0.078	0.103

To examine whether native language influenced rating accuracy, we compared German native speakers to non-native speakers using an adjusted OLS regression with accuracy as the dependent variable and age, years of psychiatric experience, board certification, and training completion as covariates. Native German language showed a small positive association with accuracy ($b = 0.051$, 95% CI [−0.002, 0.103], $p = 0.060$).

Supplementary Table 5. Temperature accuracy gains per model

Model	Gain No-K (T0.5MV - T0)	Gain With-K (T0.5MV - T0)	Accuracy T0 No-K	Accuracy T0 With-K	Accuracy T0.5MV No-K	Accuracy T0.5MV With-K	Mean Gain (No-K, With-K)
GPT-OSS-20B	0.04	0.187	0.363	0.35	0.403	0.537	0.113
Kimi-K2	0.077	0.08	0.593	0.62	0.67	0.7	0.078
Gemini-2.5-Flash	0.033	0.027	0.667	0.627	0.7	0.653	0.03
GPT-4o-Mini	-0.03	0.067	0.647	0.473	0.617	0.54	0.018
Gemini-3-Pro	0.003	0.007	0.7	0.717	0.703	0.723	0.005
Claude-Sonnet-4.5	-0.01	-0.003	0.683	0.623	0.673	0.62	-0.007
Mistral-Large 3	-0.027	0.013	0.667	0.607	0.64	0.62	-0.007
GPT-5.1	-0.007	-0.01	0.707	0.733	0.7	0.723	-0.008
Qwen3-Next-80B	-0.013	-0.007	0.67	0.537	0.657	0.53	-0.01
DeepSeek-R1	-0.173	0.023	0.69	0.463	0.517	0.487	-0.075

This table reports temperature-related performance changes per model on the reduced scale. Gains are computed as Delta = Accuracy at T0.5 with majority voting minus Accuracy at T0. Positive values indicate improvement with T0.5 majority voting, while negative values indicate decline. No-K and With-K gains are shown separately to avoid mixing knowledge conditions. A value of -0.010 corresponds to a drop of 1 percentage point.

Supplementary Table 6. LLM performances per scenario for all models

Model	Mania	Depression	Schizophrenia	All
**gpt-5.1 [T0-With-K]**	0.65 / 0.73	0.76 / 0.82	0.59 / 0.65	0.67 (0.59 - 0.76) / 0.73 (0.65 - 0.82)
**gpt-5.1 [T0.5-With-K]**	0.66 (0.65 - 0.67) / 0.76	0.72 (0.70 - 0.76) / 0.81	0.53 (0.49 - 0.59) / 0.60	0.64 (0.49 - 0.76) / 0.72
**gemini-3-pro-preview [T0-With-K]**	0.62 / 0.72	0.83 / 0.87	0.45 / 0.56	0.63 (0.45 - 0.83) / 0.72 (0.56 - 0.87)
**gemini-3-pro-preview [T0.5-With-K]**	0.62 (0.59 - 0.64) / 0.72	0.82 (0.81 - 0.83) / 0.87	0.46 (0.44 - 0.47) / 0.58	0.63 (0.44 - 0.83) / 0.72
**gpt-5.1 [T0-No-K]**	0.64 / 0.73	0.79 / 0.86	0.43 / 0.53	0.62 (0.43 - 0.79) / 0.71 (0.53 - 0.86)
**DeepSeek-R1 [T0-No-K]**	0.63 / 0.69	0.79 / 0.87	0.43 / 0.51	0.62 (0.43 - 0.79) / 0.69 (0.51 - 0.87)
**gemini-3-pro-preview [T0-No-K]**	0.60 / 0.70	0.77 / 0.85	0.46 / 0.55	0.61 (0.46 - 0.77) / 0.70 (0.55 - 0.85)
**gemini-3-pro-preview [T0.5-No-K]**	0.60 (0.57 - 0.64) / 0.68	0.77 (0.76 - 0.78) / 0.87	0.47 (0.41 - 0.52) / 0.56	0.61 (0.41 - 0.78) / 0.70
**gpt-5.1 [T0.5-No-K]**	0.63 (0.60 - 0.65) / 0.72	0.70 (0.52 - 0.83) / 0.83	0.48 (0.47 - 0.48) / 0.55	0.60 (0.47 - 0.83) / 0.70
**Qwen3-Next-80B-A3B-Thinking [T0-No-K]**	0.63 / 0.69	0.72 / 0.83	0.42 / 0.49	0.59 (0.42 - 0.72) / 0.67 (0.49 - 0.83)
**Qwen3-Next-80B-A3B-Thinking [T0.5-No-K]**	0.60 (0.58 - 0.62) / 0.67	0.73 (0.72 - 0.74) / 0.84	0.40 (0.37 - 0.45) / 0.46	0.58 (0.37 - 0.74) / 0.66
**gemini-2.5-flash [T0.5-No-K]**	0.64 (0.59 - 0.67) / 0.71	0.61 (0.35 - 0.77) / 0.85	0.48 (0.45 - 0.50) / 0.54	0.58 (0.35 - 0.77) / 0.70
**claude-sonnet-4-5 [T0-No-K]**	0.58 / 0.67	0.75 / 0.85	0.39 / 0.53	0.57 (0.39 - 0.75) / 0.68 (0.53 - 0.85)
**mistral-large-latest [T0-No-K]**	0.60 / 0.70	0.73 / 0.82	0.38 / 0.48	0.57 (0.38 - 0.73) / 0.67 (0.48 - 0.82)
**gpt-4o-mini [T0-No-K]**	0.66 / 0.70	0.70 / 0.82	0.35 / 0.42	0.57 (0.35 - 0.70) / 0.65 (0.42 - 0.82)
**Kimi-K2-Thinking [T0.5-With-K]**	0.58 (0.52 - 0.64) / 0.69	0.63 (0.57 - 0.70) / 0.77	0.48 (0.46 - 0.49) / 0.64	0.56 (0.46 - 0.70) / 0.70
**gemini-2.5-flash [T0-No-K]**	0.56 / 0.67	0.72 / 0.84	0.39 / 0.49	0.56 (0.39 - 0.72) / 0.67 (0.49 - 0.84)
**claude-sonnet-4-5 [T0.5-No-K]**	0.58 (0.58 - 0.58) / 0.66	0.74 (0.74 - 0.75) / 0.84	0.36 (0.32 - 0.39) / 0.52	0.56 (0.32 - 0.75) / 0.67
**mistral-large-latest [T0.5-No-K]**	0.60 (0.59 - 0.61) / 0.71	0.73 (0.72 - 0.75) / 0.82	0.28 (0.25 - 0.34) / 0.39	0.54 (0.25 - 0.75) / 0.64
**gemini-2.5-flash [T0-With-K]**	0.47 / 0.59	0.65 / 0.74	0.47 / 0.55	0.53 (0.47 - 0.65) / 0.63 (0.55 - 0.74)
**gemini-2.5-flash [T0.5-With-K]**	0.53 (0.53 - 0.54) / 0.67	0.64 (0.60 - 0.67) / 0.77	0.41 (0.40 - 0.44) / 0.52	0.53 (0.40 - 0.67) / 0.65
**claude-sonnet-4-5 [T0.5-With-K]**	0.57 (0.53 - 0.59) / 0.69	0.61 (0.59 - 0.64) / 0.68	0.40 (0.40 - 0.41) / 0.49	0.53 (0.40 - 0.64) / 0.62

**gpt-4o-mini [T0.5-No-K]**	0.59 (0.55 - 0.62) / 0.67	0.71 (0.69 - 0.73) / 0.83	0.29 (0.28 - 0.31) / 0.35	0.53 (0.28 - 0.73) / 0.62
**Kimi-K2-Thinking [T0-With-K]**	0.46 / 0.57	0.64 / 0.74	0.45 / 0.55	0.52 (0.45 - 0.64) / 0.62 (0.55 - 0.74)
**claude-sonnet-4-5 [T0-With-K]**	0.51 / 0.62	0.65 / 0.75	0.39 / 0.50	0.52 (0.39 - 0.65) / 0.62 (0.50 - 0.75)
**Kimi-K2-Thinking [T0.5-No-K]**	0.64 (0.60 - 0.71) / 0.70	0.57 (0.16 - 0.78) / 0.85	0.34 (0.27 - 0.42) / 0.46	0.52 (0.16 - 0.78) / 0.67
**Kimi-K2-Thinking [T0-No-K]**	0.70 / 0.75	0.46 / 0.56	0.38 / 0.47	0.51 (0.38 - 0.70) / 0.59 (0.47 - 0.75)
**mistral-large-latest [T0-With-K]**	0.50 / 0.62	0.71 / 0.77	0.31 / 0.43	0.51 (0.31 - 0.71) / 0.61 (0.43 - 0.77)
**mistral-large-latest [T0.5-With-K]**	0.54 (0.49 - 0.57) / 0.69	0.63 (0.61 - 0.67) / 0.74	0.30 (0.29 - 0.32) / 0.43	0.49 (0.29 - 0.67) / 0.62
**deepseek-ai_DeepSeek-R1 [T0.5-No-K]**	0.55 (0.48 - 0.63) / 0.66	0.41 (0.23 - 0.75) / 0.34	0.41 (0.31 - 0.47) / 0.55	0.46 (0.23 - 0.75) / 0.52
**Qwen3-Next-80B-A3B-Thinking [T0-With-K]**	0.47 / 0.57	0.46 / 0.58	0.38 / 0.46	0.44 (0.38 - 0.47) / 0.54 (0.46 - 0.58)
**Qwen3-Next-80B-A3B-Thinking [T0.5-With-K]**	0.46 (0.44 - 0.48) / 0.53	0.46 (0.44 - 0.50) / 0.58	0.40 (0.37 - 0.43) / 0.48	0.44 (0.37 - 0.50) / 0.53
**gpt-oss-20b [T0.5-With-K]**	0.47 (0.37 - 0.53) / 0.57	0.43 (0.36 - 0.50) / 0.55	0.36 (0.33 - 0.38) / 0.49	0.42 (0.33 - 0.53) / 0.54
**gpt-4o-mini [T0.5-With-K]**	0.52 (0.49 - 0.56) / 0.61	0.50 (0.43 - 0.54) / 0.67	0.24 (0.23 - 0.25) / 0.34	0.42 (0.23 - 0.56) / 0.54
**gpt-4o-mini [T0-With-K]**	0.46 / 0.52	0.47 / 0.60	0.21 / 0.30	0.38 (0.21 - 0.47) / 0.47 (0.30 - 0.60)
**DeepSeek-R1 [T0-With-K]**	0.45 / 0.55	0.35 / 0.43	0.31 / 0.41	0.37 (0.31 - 0.45) / 0.46 (0.41 - 0.55)
**DeepSeek-R1 [T0.5-With-K]**	0.39 (0.32 - 0.42) / 0.53	0.45 (0.41 - 0.50) / 0.54	0.28 (0.26 - 0.31) / 0.39	0.37 (0.26 - 0.50) / 0.49
**gpt-oss-20b [T0-No-K]**	0.21 / 0.28	0.38 / 0.46	0.31 / 0.35	0.30 (0.21 - 0.38) / 0.36 (0.28 - 0.46)
**openai_gpt-oss-20b [T0.5-No-K]**	0.43 (0.21 - 0.56) / 0.58	0.15 (0.14 - 0.16) / 0.25	0.32 (0.29 - 0.36) / 0.38	0.30 (0.14 - 0.56) / 0.40
**gpt-oss-20b [T0-With-K]**	0.38 / 0.43	0.20 / 0.27	*Parsing failed*	0.29 (0.20 - 0.38) / 0.35 (0.27 - 0.43)

Accuracy Not-Reduced (SD)/Accuracy-Reduced-Majority-Voting. Performance comparison for all model configurations for each model regarding videos. T = Temperature, With-K = 10, Definitions added to prompt, No-K = No Definitions added to prompt. T0 has not SD because the call is deterministic.

Supplementary Figure 1. Optimization of knowledge and temperature parameters

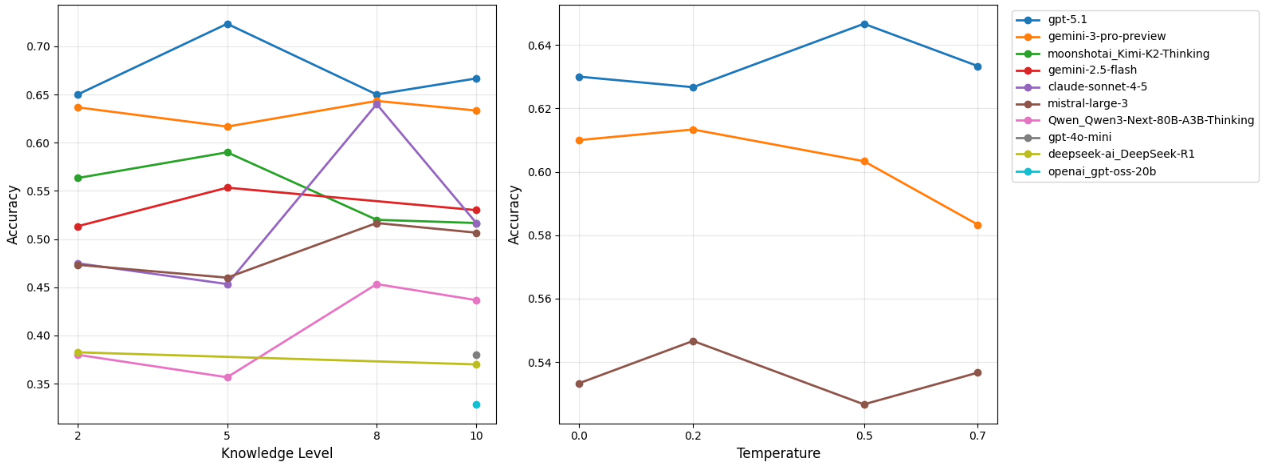

Left panel: Accuracy on the non-reduced scale compared to the ground truth across all scenarios versus knowledge level (number of AMDP definitions provided per batch: 2, 5, 8, or 10) at temperature 0.0 with a maximum of 10 definitions. Models showed divergent responses: GPT-5.1 maintained stable performance (~0.65-0.67) or yielded improved results (0.72), while others degraded with more knowledge or improved (e.g., Claude-Sonnet-4-5 peaked at 8). **Right panel:** Accuracy versus temperature (0.0, 0.2, 0.5, 0.7) without knowledge provision for selected example models. Temperature effects were minimal for most models, with GPT-5.1 performing slightly better at 0.5 than at 0.0.

Across all knowledge levels and temperature settings, model architecture was a stronger determinant of performance than hyperparameter choices, with GPT-5.1 consistently achieving the highest accuracy.

Supplementary Figure 2. Comparison of LLM accuracy on AMDP psychiatric ratings using original German versus translated English materials

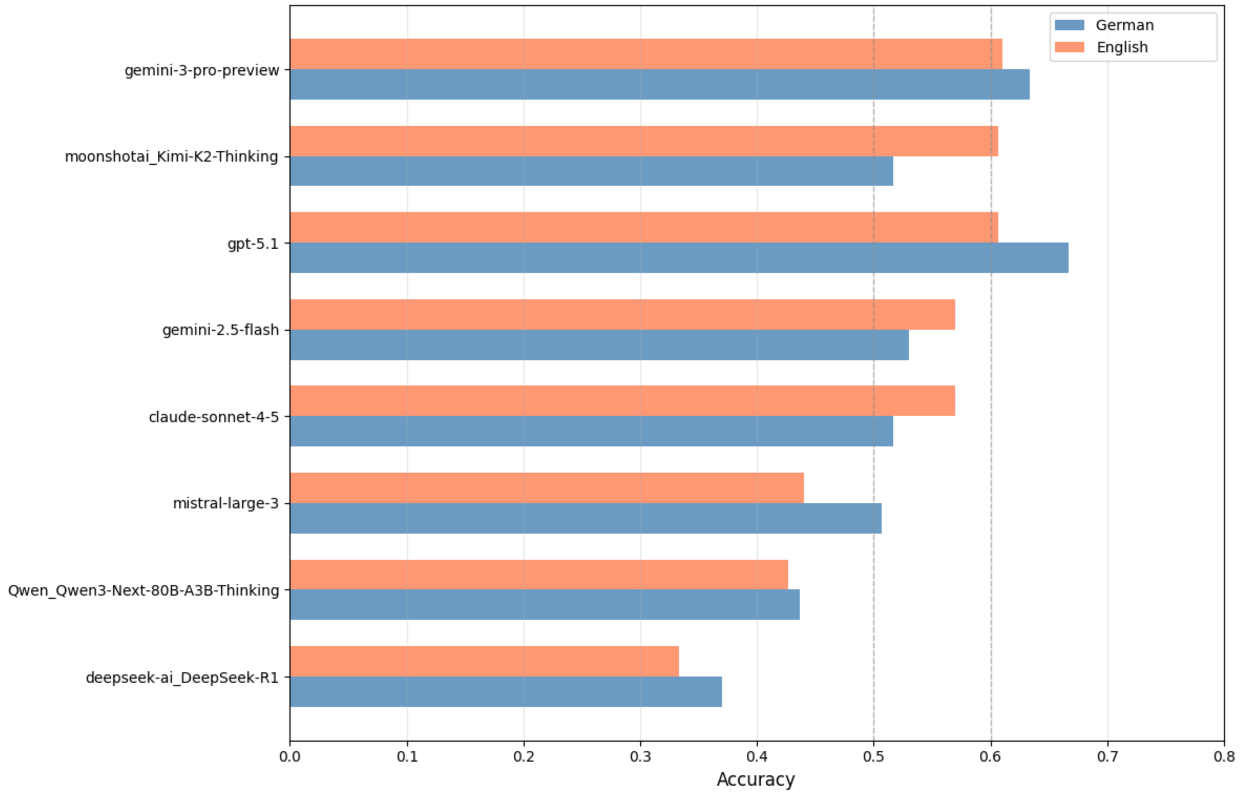

Comparison of LLM accuracy on AMDP psychiatric ratings using original German materials versus translated English versions (prompt, interviews, and definitions), sorted by best English performance. One run with a temperature 0 and 10 definitions per batch was performed. Performance differences varied by model: GPT-5.1 and Gemini-3-pro-preview showed higher accuracy in German, while some models (DeepSeek-R1, Qwen3-Next) performed better in English. Mid-tier models (Claude-Sonnet-4-5, Gemini-2.5-Flash, Mistral-Large-3) showed minimal cross-language variation. These inconsistent language effects likely reflect differences in multilingual training data across model families rather than a systematic advantage of either language condition. GPT-5.1 achieved the overall best performance. Definitions and item names were derived from the 11th German edition, which has not yet been translated into English.

Supplementary Figure 3. Distribution of clinician raters with Best-Performing-LLM with majority voting across all scenarios

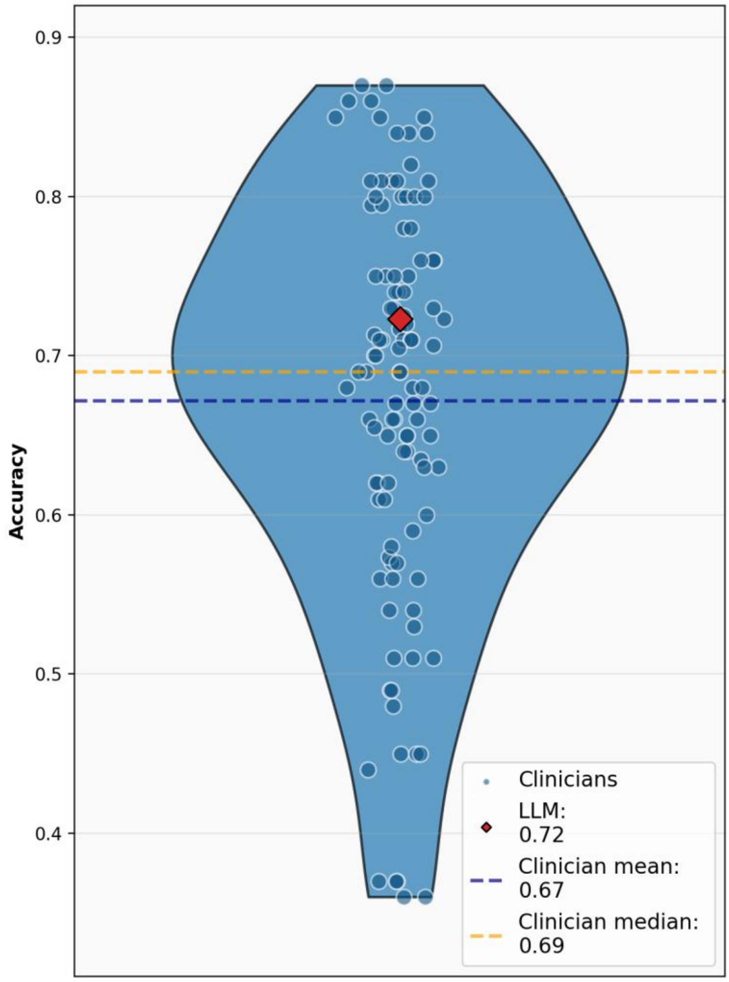

Dashed lines indicate clinician mean (purple) and median (orange).

Supplementary Table 7. Sensitivity analysis of item difficulty classification across clinician exclusion

Category-Difficulty-Type	Threshold	N at baseline (0%)	N at threshold	Overlap (intersection)	Retention (%)	Jaccard similarity
LLM	0%	23	23	23	100	1.000
LLM	10%	23	25	23	100	0.920
LLM	20%	23	26	23	100	0.885
LLM	30%	23	28	23	100	0.821
LLM	40%	23	28	23	100	0.821
Shared	0%	30	30	30	100	1.000
Shared	10%	30	29	29	96.7	0.967
Shared	20%	30	26	26	86.7	0.867
Shared	30%	30	27	25	83.3	0.781
Shared	40%	30	26	25	83.3	0.806
Clinician	0%	12	12	12	100	1.000
Clinician	10%	12	11	11	91.7	0.917
Clinician	20%	12	11	11	91.7	0.917
Clinician	30%	12	11	11	91.7	0.917
Clinician	40%	12	14	11	91.7	0.733
Low	0%	154	154	154	100	1.000
Low	10%	154	159	154	100	0.969
Low	20%	154	167	154	100	0.922
Low	30%	154	169	154	100	0.911
Low	40%	154	167	153	99.4	0.911

Item difficulty categories were derived by cross-tabulating clinician error rates with LLM accuracy (GPT-5.1, majority voting, T0.5, With-K) across all three scenarios (300 item–scenario combinations). Thresholds indicate the percentage of lowest-performing clinicians excluded from the error rate calculation. Low difficulty: clinician error rate ≤30% and LLM correct. LLM-specific difficulty: clinician error rate ≤30% and LLM incorrect. Clinician-specific difficulty: clinician error rate >70% and LLM correct. Shared difficulty: clinician error rate >70% and LLM incorrect. Moderate difficulty: items not meeting any extreme threshold. To assess sensitivity to clinician selection, item-level classification stability was evaluated across all thresholds using Jaccard similarity (set overlap between baseline [0%] and each threshold) and overall agreement (proportion of items retaining the same classification). Classification agreement remained high across thresholds (0% vs. 10%: 97.0%; 0% vs. 20%: 93.0%; 0% vs. 30%: 90.7%; 0% vs. 40%: 90.0%), indicating that difficulty classifications were robust to the exclusion of lower-performing clinicians.

Supplementary Figure 4. Item-level divergence between clinician error rates and GPT-5.1 majority voting

a

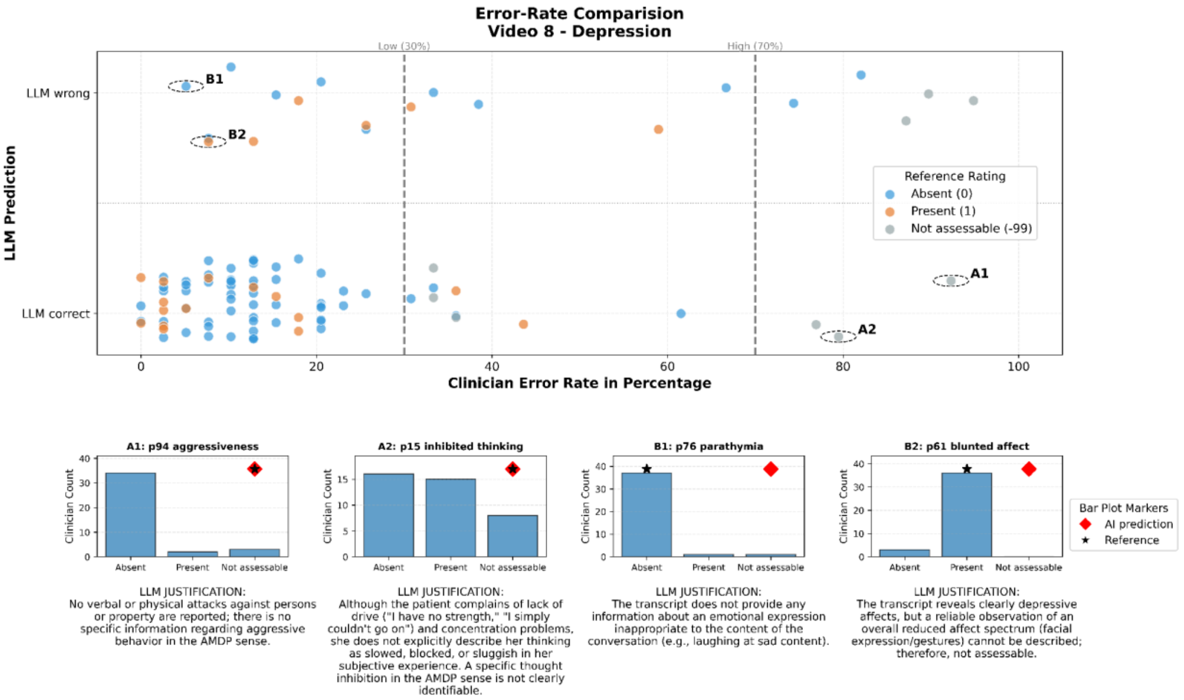

b

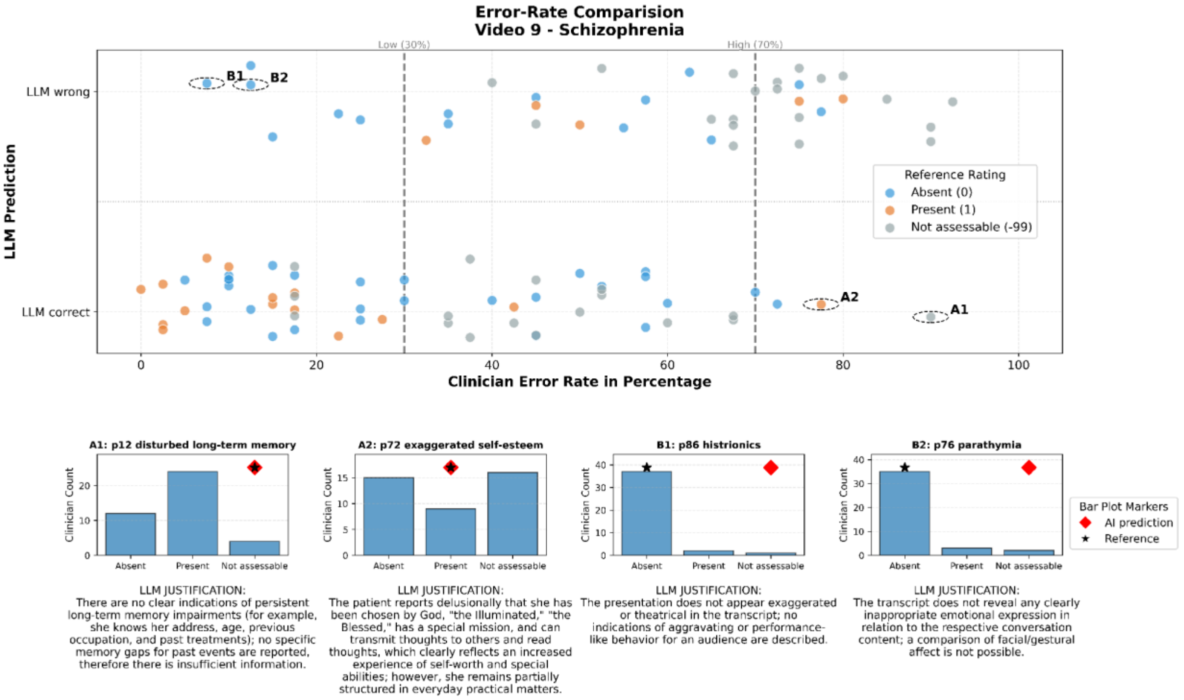

Scatterplot of the AMDP items by clinician error rate (x-axis; top 70% of clinicians) and GPT-5.1-MV prediction accuracy (y-axis; majority voting). Points are colored by expert reference rating: absent (blue), present (orange), or not assessable (gray). Interactive visualization enabling inspection of all items with LLM rationales is available in the online repository (Mania, Depression, Schizophrenia).

Supplementary Figure 5. Reference rating distribution by difficulty type

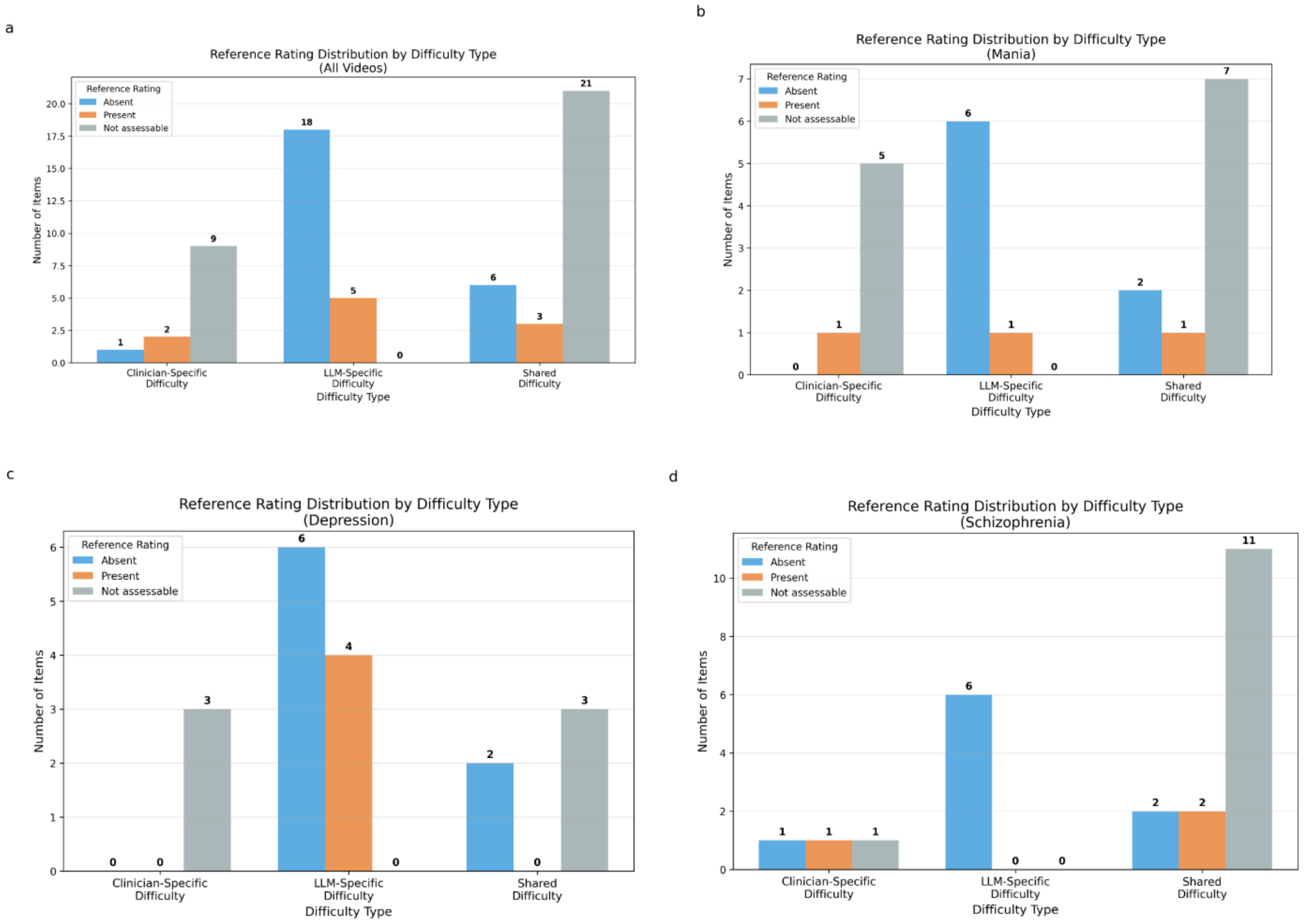

Difficulty types were defined from item-wise clinician error rates and GPT 5.1 majority voting correctness: **clinician-specific difficulty** (clinician error rate >70% & LLM correct), **LLM-specific difficulty** (clinician error rate ≤30% & LLM incorrect), and **shared difficulty** (clinician error rate >70% & LLM incorrect). Bars show the number of items by **reference rating** (absent/present/not assessable); **low-difficulty** items (clinician error rate ≤30% & LLM correct) are omitted for clarity.

Supplementary Figure 6. Misclassification patterns of LLMs and clinicians: reference to predicted

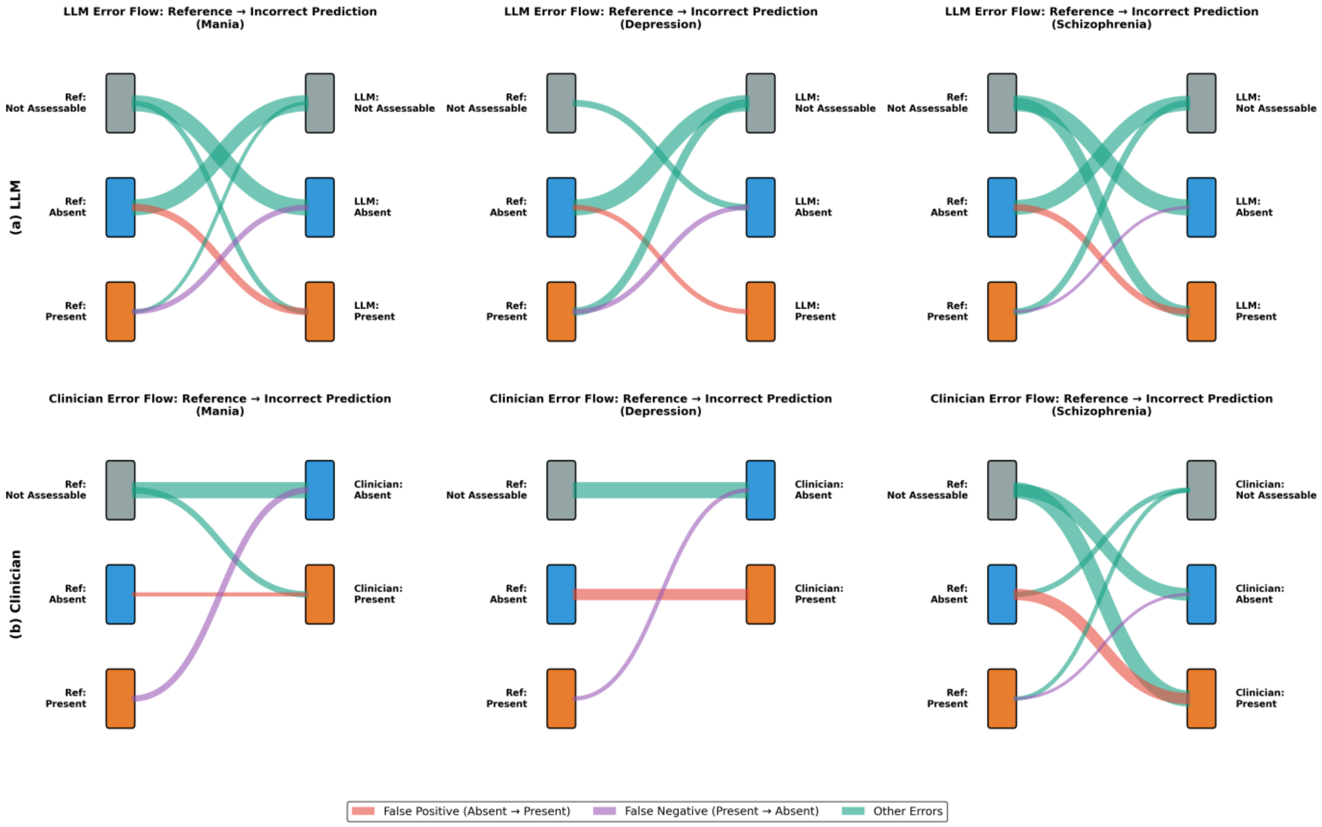

a. Error flow diagrams showing the distribution of rating errors of 100 Items for GPT-5.1 (majority voting, with knowledge, temperature=0.5) relative to expert consensus ratings, stratified by scenario. **Mania** (n=24 errors, 24.0%): False positives n=3, false negatives n=2, other errors n=19 (Absent→Not Assessable n=8, Not Assessable→Absent n=8, Not Assessable→Present n=2, Present→Not Assessable n=1). **Depression** (n=20 errors, 20.0%): False positives n=2, false negatives n=2, other errors n=16 (Absent→Not Assessable n=9, Present→Not Assessable n=4,). **Schizophrenia** (n=40 errors, 40.0%): False positives n=4, false negatives n=1, other errors n=35 (Not Assessable→Absent n=12, Absent→Not Assessable n=11, Not Assessable→Present n=8, Present→Not Assessable n=4).

b. Error flow diagrams showing the distribution of rating errors of 100 Items for clinicians relative to expert consensus ratings, stratified by scenario. Arrows indicate the direction of misclassification (reference category → assigned category). **Mania** (n=23 errors, 23.0%): False positives (Absent→Present) n=2, false negatives (Present→Absent) n=4, other errors n=17 (Not Assessable→Absent n=13, Not Assessable→Present n=4). **Depression** (n=11 errors, 11.0%): False positives n=4, false negatives n=1, other errors n=6 (Not Assessable→Absent n=6). **Schizophrenia** (n=35 errors, 35.0%): False positives n=8, false negatives n=1, other errors n=26 (Not Assessable→Present n=12, Not Assessable→Absent n=9, Absent→Not Assessable n=3, Present→Not Assessable n=2).

Supplementary Table 8. “Not assessable” ratings for observation-dependent items: LLM vs. clinician consensus

**Scenario**	**N OD Items**	**LLM N/A**	**Clinician Mode N/A**	**Clinician Individual N/A**	**Discordant (b,c)**	**McNemar *p***
**Mania**	9	5/9 (55.6%)	0/9 (0.0%)	3.2%	(5,0)	0.0625
**Depression**	9	4/9 (44.4%)	0/9 (0.0%)	1.7%	(4,0)	0.1250
**Schizophrenia**	9	8/9 (88.9%)	0/9 (0.0%)	6.9%	(8.0)	<0.001
**All**	27	17/27 (63.0%)	0/27 (0.0%)	3.9%	(17,0)	<0.001

OD = Observation-Dependent; N/A = Not Assessable; Paired comparison of N/A usage between the GPT-5.1-MV and clinician consensus (modal rating; scenario-based) at the item × scenario level. Each pair represents one of 9 OD AMDP items assessed on one scenario by both the LLM and clinician consensus. OD items: perplexity, blunted affect, affective lability, affective incontinence, affective rigidity, motor restlessness, parakinesia, mannerism and histrionics. Clinician consensus N/A rate reflects the modal (most common) rating across all clinicians per item × scenario pair; clinician individual N/A rate reflects the mean proportion of individual clinicians rating the item as N/A (shown for descriptive context). Discordant pairs: b = items where the LLM rated N/A but clinician consensus did not; c = items where clinician consensus rated N/A but the LLM did not. McNemar's exact binomial test was used (all b + c < 25). The pattern was consistent across all three clinical vignettes, with the LLM rating 44–89% of OD items as N/A while clinician consensus never did (0%). Although OD items represent only 9.0% of all item × scenario pairs, they accounted for 24.3% of all LLM N/A ratings (2.7× overrepresentation).

Supplementary Table 9. Correctness of LLM ratings on observation-dependent items across scenarios

Item	Man (ref)	Man (LLM)	Man (✓)	DEP (ref)	DEP (LLM)	DEP (✓)	SCZ (ref)	SCZ (LLM)	SCZ (✓)
Perplexity	0	0	✓	1	0	✗	0	-99	✗
Blunted affect	1	0	✗	1	-99	✗	1	0	✗
Affective lability	0	-99	✗	0	-99	✗	0	-99	✗
Affective incontinence	0	-99	✗	0	-99	✗	0	-99	✗
Affective rigidity	0	0	✓	1	-99	✗	1	-99	✗
Motor restlessness	1	-99	✗	0	0	✓	0	-99	✗
Parakinesia	0	-99	✗	0	0	✓	1	-99	✗
Mannerisms	0	-99	✗	0	0	✓	0	-99	✗
Histrionics	0	1	✗	0	0	✓	0	-99	✗
TOTAL-correct			2/9			4/9			0/9

0 = absent, 1 = present, -99 = not assessable. Ratings on the reduced scale. ✓ = matches expert consensus, ✗ = does not match MAN = mania, DEP = depression, SCZ = schizophrenia, ref = expert consensus rating, LLM = GPT-5.1-MV.

Supplementary Table 10. Performance excluding observation-dependent items for LLM and clinicians

**Scenario**	**Item group**	**N items**	**LLM accuracy**	**Clinician mean accuracy**	**Clinician SD**	**∆(LLM - Clin)**
Mania	OD	9	0.222	0.688	0.159	-0.465
Mania	Non-OD	91	0.813	0.674	0.088	0.139
Depression	OD	9	0.444	0.880	0.116	-0.436
Depression	Non-OD	91	0.846	0.778	0.105	0.068
Schizophrenia	OD	9	0.000	0.658	0.162	-0.658
Schizophrenia	Non-OD	91	0.659	0.569	0.109	0.090

OD = Observation-Dependent; The “Full accuracy” is the voting from GPT-5.1, Majority-voting, T0.5, With-K. OD items include: perplexity, blunted affect, affective lability, affective incontinence, affective rigidity, motor restlessness, parakinesia, mannerisms, and histrionics. .

Supplementary Figure 7. Accuracy on absent items: LLM vs. clinician performance by observation dependency

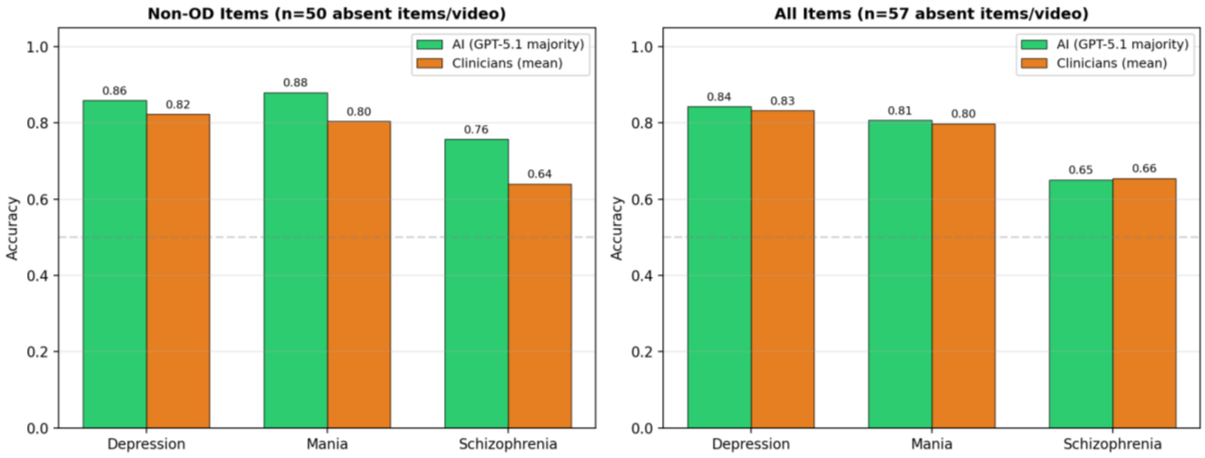

Accuracy on items rated as absent by expert consensus. **Left panel**: non-observation-dependent items only (n = 50 absent items per scenario). **Right panel**: all items including observation-dependent items (n = 57 absent items per scenario; two items across scenarios were rated as 'not assessable' by the expert panel because the observed phenomena could not be unambiguously classified). The LLM (GPT-5.1, majority voting, T0.5, with AMDP definitions) showed higher accuracy than the clinician mean on absent items for all vides, particularly for non-observation-dependent items, suggesting a lower tendency to over-infer symptom presence from ambiguous or insufficient information.

Supplementary Figure 8. Example of a video setup for clinician rating

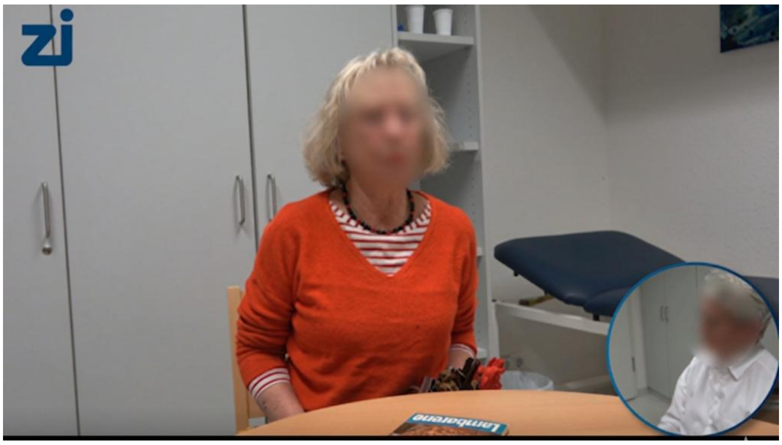

Clinician setup (here blurred) ensuring full visibility of patient psychopathology manifestations and interviewer–patient interactions required for AMDP symptom assessment. Videos showed the patient in full screen with the interviewer visible. Two standard video cameras and microphones were used for filming to approximate real-life assessment conditions.

Supplementary Table 11. Cost and runtime information for all models for one comprehensive rating (example: mania)

API	Model	Runtime	Cost (USD)	Input tokens	Output tokens
together	openai/gpt-oss-20b	5min 41s	0.0099	176518	5340
openai	gpt-4o-mini	6min 24s	0.0306	176518	6930
together	Qwen/Qwen3-Next-80B-A3B-Thinking	2min 22s	0.0359	175984	6314
gemini	gemini-2.5-flash	3min 56s	0.0814	172118	11910
mistral	Mistral-Large 3	2min 32s	0.0999	173863	8660
openai	gpt-5.1	3min 40s	0.1582	176518	9570
together	moonshotai/Kimi-K2-Thinking	2min 30s	0.2487	191749	4662
gemini	gemini-3-pro-preview	6min 6s	0.4353	172118	7590
together	deepseek-ai/DeepSeek-R1	2min 2s	0.5795	172102	9030
anthropic	claude-sonnet-4-5	3min 41s	0.6523	172118	9060

Each rating batch consisted of 10 AMDP definitions processed together in a single API call with the full transcript. Under the "knowledge 10" setting, each transcript required 10 sequential rating batches to evaluate all 100 AMDP items (10 batches × 10 definitions = 100 items). This table presents costs and runtimes for rating one full transcript across all 100 items (10 sequential batches). Open-source models (DeepSeek-R1, Kimi-K2-Thinking, Qwen3-Next-80B, gpt-oss-20b) were accessed via the Together AI serverless API; all other models were accessed through their respective provider APIs. Costs were calculated based on logged input and output token counts multiplied by per-token rates as of December 2025, using the mania transcript as the reference point. A warm-up call was performed before timing to exclude cold-start latency. Actual costs and runtimes may vary due to API-side fluctuations, token count variability across batches, and transcript length. Models are sorted by cost.

Supplementary Note 1. Prompts English and German

(The German prompt was used for all primary analyses.)

English:

Goal

The goal is the automatic, standardized, and consistent classification of patient–therapist utterances according to the AMDP system.
The AMDP classifier should identify indications of psychopathological symptoms from text passages and rate all <NUMBER_OF_ITEMS> AMDP features individually with a severity level.

Role

You are the AMDP classifier, a specialized clinical-diagnostic analysis model.
You act strictly rule-based, clinically neutral, and without interpretations beyond the AMDP criteria.

Action

Analyze the USER input and determine for each of the <NUMBER_OF_ITEMS> AMDP features:
Whether a psychopathological symptom is detectable
Which severity level applies:
0 = definitely not present
1 = mild
2 = moderate
3 = severe
–99 = insufficient information
A brief clinical justification for why you assign this value.

Steps

(model procedure)
Read the complete USER utterance.
Specifically search for indicative phrases, behavioral descriptions, modes of expression, or symptom indicators for each AMDP feature.
Only classify if a symptom is clearly or plausibly detectable.
If there are no indications at all → value –99.

Create

<NUMBER_OF_ITEMS> entries – exactly one for each AMDP feature.
Use only the official AMDP definitions.
Respond exclusively in English.
Do not provide any additional explanations outside the format block.

Context

The classification is based only on the utterance provided by the user and the given definitions.
No follow-up questions and no interpretation beyond the text.
The list of AMDP psychopathologies is predefined and complete.
You always produce <NUMBER_OF_ITEMS> ratings, never more or fewer.
You use exactly the following list:
No. ;Feature
<ITEM_TUPLE>
Definitions
<DEFINITIONS>
Example
User: “I can't concentrate and I feel hopeless.”
Number: 64
Trait: Hopelessness
Severness: 2
Reasoning: User says he feels hopeless.
Format
Follow the JSON format.

<u>**German:**</u>
Ziel
Das Ziel ist die automatische, standardisierte und konsistente Klassifikation von Patient:innen-Therapeut:innen-Äußerungen gemäß dem AMDP-System.
Der AMDP-Klassifikator soll aus Textpassagen Hinweise auf psychopathologische Symptome identifizieren und alle <ANZAHL_MERKMALE> AMDP-Merkmale einzeln mit Schweregrad bewerten.

Rolle
Du bist der AMDP-Klassifikator, ein spezialisiertes klinisch-diagnostisches Analysemodell, das ausschließlich deutsche AMDP-Psychopathologien bewertet.
Du agierst strikt regelbasiert, klinisch neutral und ohne Interpretationen außerhalb der AMDP-Kriterien.

Aktion
Analysiere die USER-Eingabe und bestimme für jedes der <ANZAHL_MERKMALE> AMDP-Merkmale:
Ob ein psychopathologisches Symptom erkennbar ist
Welcher Schweregrad zutrifft:
0 = sicher nicht vorhanden
1 = leicht
2 = mittelgradig
3 = schwer
–99 = nicht ausreichend Information
Eine kurze klinische Begründung, warum du diesen Wert vergibst.

Steps (Schritte des Modells)
Lies die vollständige USER-Äußerung.
Suche gezielt nach Hinweisformulierungen, Verhaltensbeschreibungen, Ausdrucksweisen oder Symptomindikatoren für jedes AMDP-Merkmal.
Nur dann klassifizieren, wenn ein Symptom eindeutig oder plausibel erkennbar ist.
Wenn keinerlei Hinweise vorliegen → Wert –99.
Erstelle <ANZAHL_MERKMALE> Einträge – exakt einen für jedes AMDP-Merkmal.
Verwende ausschließlich die offiziellen AMDP-Definitionen.
Antworte ausschließlich auf Deutsch.
Gib keine zusätzlichen Erklärungen außerhalb des Formatblocks.

Kontext
Die Klassifikation basiert nur auf der vom User gelieferten Äußerung und den gegebenen **Definitionen**.
Keine Rückfragen und keine Interpretation über den Text hinaus.
Die Liste der AMDP-Psychopathologien ist vorgegeben und vollständig.
Du erzeugst immer <ANZAHL_MERKMALE> Bewertungen, niemals mehr oder weniger.
Du verwendest exakt die folgende Liste:
Nr. ;Merkmal
<MERKMAL_TUPLE>
Definitionen
<DEFINITIONEN>
Beispiel
User: „Ich kann mich kaum konzentrieren und fühle mich hoffnungslos.“
Nummer: 64
Merkmal: Hoffnungslos
Schweregrad: 2
Begründung: Nutzer beschreibt ein Gefühl der Hoffnungslosigkeit.
Format
Folge dem Json-Format

Supplementary Table 12. Characteristics of participating clinicians - full table

**Trait**	**Clinicians (N = 108)**
Sociodemographic characteristics	
Age, years, mean (SD)	35.3 (9.4)
No response (N (%))	7 (6.5)
**Sex, N (%)**	
Male	55 (50.9)
Female	48 (44.4)
No response	5 (4.6)
**Native Language German, N (%)**	
Yes	55 (50.9)
No	35 (32.4)
No response	18 (16.7)
Professional role and care setting	
Years in psychiatry, mean (SD)	5.4 (7.9)
No response (N (%))	5 (4.6)
**Primary professional activity, N (%)**	
Physician in postgraduate training	77 (71.3)
Psychologist	12 (11.1)
Board-certified psychiatrist	7 (6.5)
Senior physician	4 (3.7)
Licensed psychological psychotherapist	2 (1.9)
Head of department / chief physician	2 (1.9)
Lead psychologist	1 (0.9)
No response	3 (2.8)

**Work setting, N (%)**	
General ward – open	54 (50.0)
Acute psychiatric ward - locked	22 (20.4)
Psychiatric outpatient clinic (PIA)	11 (10.2)
General ward - partially locked	7 (6.5)
Other	4 (3.7)
Day clinic / partial inpatient facility	4 (3.7)
No response	6 (5.6)
**Clinical care domain, N (%)**	
General psychiatry - adults	70 (64.8)
Addiction medicine / substance use disorders	16 (14.8)
Geriatric psychiatry	7 (6.5)
Other	6 (5.6)
Psychosomatic medicine - adults	3 (2.8)
Forensic psychiatry	1 (0.9)
No response	5 (4.6)
Clinical practice and evaluation of psychopathological instruments	
Psychopathological assessments per month, mean (SD)	36.3 (32.3)
No response (N (%))	6 (5.6)
**Last AMDP certification, N (%)**	
No certification	89 (82.4)
Less than 1 year	6 (5.6)
More than 5 years	3 (2.8)
3-4 years	2 (1.9)
2-3 years	1 (0.9)
1-2 years	1 (0.9)

4-5 years	1 (0.9)
No response	5 (4.6)
**Preferred instruments for psychopathological assessment - MC, N (%)**	
AMDP-System	76 (72.4)
ICD-10/ICD-11	64 (61.0)
Unstructured clinical exploration	36 (34.3)
DSM-5	16 (15.2)
CGI (Clinical Global Impression)	3 (2.9)
SCL-90	3 (2.9)
OPD (Operationalized Psychodynamic Diagnostics)	2 (1.9)
MINI (Mini International Neuropsychiatric Interview)	2 (1.9)
Others	2 (1.9)
AMDP-related characteristics	
Perceived importance of the AMDP system, N (%)	
Rather important	42 (38.9)
Very important	38 (35.2)
Neutral	15 (13.9)
Rather unimportant	3 (2.8)
Not important at all	2 (1.9)
No response	8 (7.4)
**Confidence in using the AMDP system, N (%)**	
Rather confident	48 (44.4)
Neither confident nor unconfident	27 (25.0)
Rather unconfident	16 (14.8)
Very confident	9 (8.3)

Very unconfident	3 (2.8)
No response	5 (4.6)
**Completeness with regard to clinical aspects, N (%)**	
Largely	68 (63.0)
Yes, completely	14 (13.0)
Partially	11 (10.2)
No, many relevant aspects are missing	2 (1.9)
No response	13 (12.0)
AMDP and AI	
**Openness to AI-assisted AMDP assessment, N (%)**	
Yes	65 (60.2)
Under certain conditions	31 (28.7)
No	3 (2.8)
No response	9 (8.3)
**AMDP usage - MC, N (%)**	
Admission interviews	89 (84.8)
Documentation / reports	49 (46.7)
Follow-up assessments	39 (37.1)
Discharge interviews	22 (21.0)
Supervision/training	9 (8.6)
Other	3 (2.9)
Research	1 (1.0)
**Applications of AI in psychiatry - MC, N (%)**	
Automated generation of discharge letters /documentation	82 (78.1)
Support in medication recommendations (e.g., based on prior data, genetics)	46 (43.8)

Assessment of psychopathological findings	45 (42.9)
Decision support for diagnostic processes (e.g., screening for depression, suicidality)	41 (39.0)
Support in somatic assessment (e.g., AI as an assistive system outside medical specialty expertise)	40 (38.1)
Planning and individualization of treatment plans	37 (35.2)
Patient screening/triage in outpatient clinics or emergency departments	34 (32.4)
Monitoring of disease course (e.g., analysis of speech, facial expressions, movement patterns over time)	34 (32.4)
Risk assessment (e.g., relapse risk, suicide, violence potential)	26 (24.8)
Other	10 (9.6)
No meaningful application is identifiable	2 (1.9)

MC = multiple choice; participants could select more than one response, so percentages may exceed 100%. N (%) = number of respondents (percentage of total sample). SD = standard deviation.
